# Ability of Lewis Acids with Shallow σ-Holes to Engage in Chalcogen Bonds in Different Environments

**DOI:** 10.3390/molecules26216394

**Published:** 2021-10-22

**Authors:** Rafał Wysokiński, Wiktor Zierkiewicz, Mariusz Michalczyk, Steve Scheiner

**Affiliations:** 1 Faculty of Chemistry, Wrocław University of Science and Technology, Wybrzeże Wyspiańskiego 27, 50-370 Wrocław, Poland; mariusz.michalczyk@pwr.edu.pl; 2 Department of Chemistry and Biochemistry, Utah State University, Logan, UT 84322-0300, USA

**Keywords:** molecular electrostatic potential, solvent effects, AIM, LMOEDA

## Abstract

Molecules of the type XYT = Ch (T = C, Si, Ge; Ch = S, Se; X,Y = H, CH_3_, Cl, Br, I) contain a σ-hole along the T = Ch bond extension. This hole can engage with the N lone pair of NCH and NCCH_3_ so as to form a chalcogen bond. In the case of T = C, these bonds are rather weak, less than 3 kcal/mol, and are slightly weakened in acetone or water. They owe their stability to attractive electrostatic energy, supplemented by dispersion, and a much smaller polarization term. Immersion in solvent reverses the electrostatic interaction to repulsive, while amplifying the polarization energy. The σ-holes are smaller for T = Si and Ge, even negative in many cases. These Lewis acids can nonetheless engage in a weak chalcogen bond. This bond owes its stability to dispersion in the gas phase, but it is polarization that dominates in solution.

## 1. Introduction

The venerable Lewis theory of acids and bases is the foundation for understanding the nature of noncovalent interactions between molecules. Interactions based on the charge transfer between interacting molecules are the essence of common and important interactions in both biological and chemical areas. Such interactions are the domain of the elements of the main groups of the periodic table. The bonding terms triel (Group 13), tetrel [1,2,3] (Group 14), pnicogen [4,5] (Group 15), chalcogen [6,7] (Group 16), halogen [8,9] (Group 17), or aerogen (noble gas) atoms (Group 18) are used for elements that act as Lewis acids. Quite recently, a similar type of interaction has been studied for Group 2 elements [10] and for the transition metals of Groups 11 and 12 [11,12]. Consistent with this, as for the H bond [13], halogen [14] and chalcogen [15] bonds, their origins and properties, have been formally defined by IUPAC.

The change from isotropic charge distributions of free atoms to anisotropic when involved in covalent bonding generates areas of depleted electron density, as well as accumulations. These depleted areas, depending on their location, are referred to as σ-holes [16,17,18,19,20] (density reduction along covalent bond extension), or π-holes [16,21,22] (above the plane of the molecule). A local reduction in electron density is usually accompanied by a positive electrostatic potential. According to the tenets of Lewis theory, acceptor and donor (lone pair, anion, π-electrons) can interact with one another to create a molecular complex which is stabilized by electrostatics, orbital interactions, and dispersion.

In contrast to the typical situation, in certain situations density depletion is not sufficient to impart a positive sign to the MEP; the hole is simply less negative when compared to other areas of the molecule. Despite this negative potential, a hole of this type can still be capable of engaging in a stabilizing interaction with an approaching nucleophile. For one thing, the specific interactions of the hole are not necessarily representative of the entire Coulombic interaction of the two monomers in their entirety. Another factor allowing such an attractive interaction is the set of polarizations within the two molecules as they approach one another. For example, one sort of density redistribution might lead to the induction of a positive hole, whereas the hole in the unperturbed monomer could be negative. It is also necessary to factor in charge transfer stabilization, and the ubiquitous attractive nature of dispersion forces. Some current literature bolsters these ideas with examples of stable halogen and chalcogen bonded complexes which were formed despite a negative σ-hole [23,24,25] in the Lewis acid molecule.

The present work is devoted to a deeper analysis of the forces that allow, or disallow, the formation of stable complexes when the hole on the Lewis acid is of negative sign. Does such a negative hole always lead to an overall repulsive electrostatic interaction with a nucleophile? How large a negative hole can be tolerated and be countered by other attractive forces to permit the formation of a stable complex? This work also explores the ability of solvation to influence the central questions: Does solvation affect the magnitude of the hole or permit a complex to form that would otherwise be unstable in vacuo? Is the overall Coulombic interaction necessarily repulsive if the Lewis acid contains a negative hole, and how large are the roles played by polarization and dispersion, not only in a vacuum but within the context of a solvent?

**Scheme 1:**
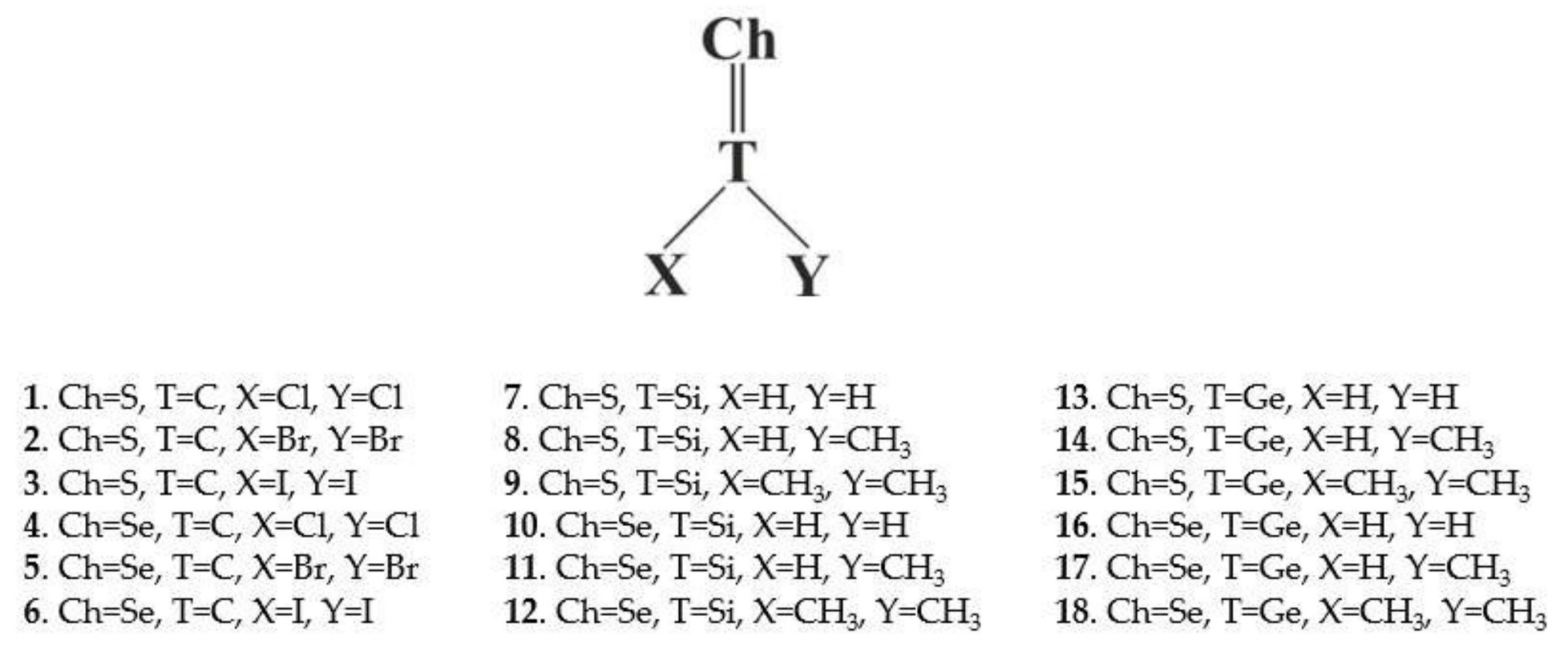
Lewis acids considered in this work.

In order to address these questions, the work applies ab initio quantum chemical calculations to a series of potentially chalcogen-bonding systems. As Lewis acids, the S and Se chalcogen atoms are attached via a double bond to a central tetrel (T) atom C, Si, or Ge. The X and Y substituents on the XYT=Ch molecule are taken from the list H, CH_3_, Cl, Br, or I. Systems containing a chalcogen atom double-bonded to a tetrel atom have been discussed in the literature [26,27]. The full list of 18 molecules considered are presented in Scheme 1. The cyano group in both N≡CH and N≡CMe were taken as the partner nucleophile, for a total of 36 different dimers considered.

## 2. Results

### 2.1. Monomers

Scheme 1 shows the Lewis acid monomers selected for study and their numerical label. These systems comprise a wide range of atom choices. As a central T tetrel atom, C was considered along with Si and Ge. The chalcogen Ch atom to which it is double-bonded was either S or Se, and the two substituents on the T atom, X and Y were a halogen Cl, Br, or I and H or CH_3_.

**Figure 1:**
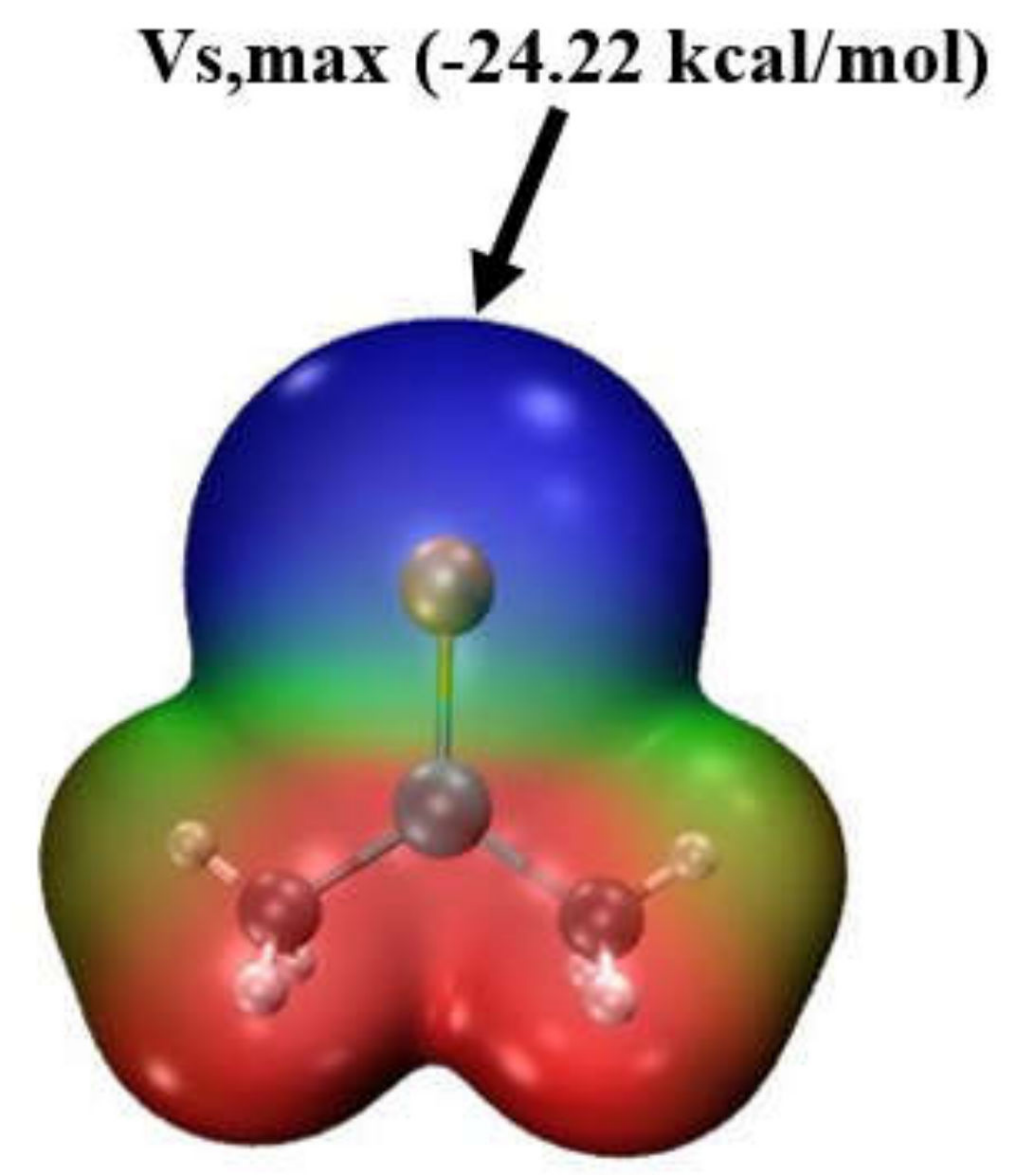
The molecular electrostatic potential map for isolated monomer **18,** (CH_3_)_2_GeSe in water solvent geometry, indicated on the contour of 0.001 au electron isodensity. Colour scale is from −0.03 au (blue) to +0.03 au (red).

The MEP surrounding each monomer is illustrated in Figure 1 using Lewis acid **18,** (CH_3_)_2_GeSe, as an example. This potential is drawn on a 0.001 au isodensity surface, and blue and red regions indicate negative and positive potentials, respectively. The blue area surrounding the Se represents visually an overall negative potential. However, there are, nonetheless, least negative points on this surface, referred to as maxima. For this particular molecule, this maximum lies along an extension of the T-Ch axis and the value of the potential at this maximum, V_s,max_, and equated to what is termed a σ-hole, is equal to −24.22 kcal/mol.

The sign of the potential at this σ-hole presents a clear differentiation between the set of molecules. For molecules **1**–**6**, i.e., all those for which T = C, V_s,max_ is positive, whereas for those with a Si or Ge tetrel atom, the σ-hole is negative. This differentiation is obvious for the first column of Table 1 which lists V_s,max_ in the gas phase. The following columns make it clear that the sign of V_s,max_, and even its numerical value, changes little when the molecule is immersed in a solvent, whether acetone or water. The largest solvent effects occur for molecules **7**–**18** for which the σ-hole becomes more negative. Note that there is little further change upon raising the dielectric constant from 20 for acetone up to 78 for water.

The last two rows of Table 1 contain the minimum in the MEP of the two bases NCH and NCMe. These minima occur on the N atom, along the extension of the N≡C bond. These values of V_s,min_ are quite negative, more so for the stronger NCMe base. As for the acids, the immersion in solvent makes these extrema more negative.

Of course, there are isodensity surfaces other than ρ = 0.001 on which one might measure the MEP. This density was chosen some years ago as a standard which might best mimic the vdW radius of each atom. The vdW radii of S and Se are equal to 1.89 and 1.82 Å, respectively. However, as may be seen in the appropriate columns of Table 1, the ρ = 0.001 au maxima are located further from these atoms by some 0.2–0.4 Å. The implications of this longer distance are discussed below.

### 2.2. Dimers

#### 2.2.1. Carbon-Containing Acids with Positive σ-Hole

Given the natural categorization of the Lewis acids into those with positive and those with negative σ-holes, it would seem sensible to consider each group separately. The two different bases were each allowed to engage in a complex with the various C-containing Lewis acids **1–6**. The notations a and b are used to differentiate dimers involving a) NCH and b) NCMe. In each of these complexes, the N atom of the base fits into a location squarely along the C=Ch axis, coincident with the alignment of the σ-hole. AIM molecular diagrams contained in Figure S1 (Supplementary Materials) indicate that these complexes are bound principally by a Ch···N chalcogen bond. The intermolecular R(Ch···N) distances are displayed in Table 2 for gas-phase as well as solvents. The first point to make about these distances is that they are considerably shorter than the sum of vdW radii (3.55 Å for N∙∙∙S and 3.48 Å N∙∙∙Se). Although these distances grow slightly longer in solvent, this perturbation is a minor one.

Consistent with the intermolecular distances shorter than the vdW radius sum, the interaction energies of all these complexes are negative, as compiled in Table 3.

These quantities vary between 1.3 and 2.4 kcal/mol in the gas phase so would fall into the category of a weak chalcogen bond. Note that the stronger NCMe base results in a somewhat stronger interaction than NCH in all cases. Placement of the complexes in solvent weakens these noncovalent bonds but all interaction energies remain negative, nonetheless. There is a strong correlation between these interaction energies and the depths of the σ-holes. The correlation coefficient R^2^ for a linear relation is 0.89, for either NCH or NCMe. The AIM descriptors of the chalcogen bonds in Tables S1–S3 are consistent with their designation as a weak noncovalent bond. The bond critical point densities are all equal to roughly 0.01 au, with a small positive ∇^2^ρ, and H close to zero.

As shown by the results in Table 4 of the LMOEDA decomposition of the total interaction within the gas phase, the largest contributors to the stabilization of these systems are the electrostatic (ES) and dispersion (DISP) terms, followed by a much smaller polarization (POL) component. DISP grows to the same scale as ES for complexes involving monomers **2, 3, 5,** and **6** (Scheme 1) where the heavier Br and I halogen atoms replace the lighter Cl of **1** and **4**. The situation changes dramatically when the dimers are immersed in solvent. As seen in the first column of Table 5, the electrostatic interaction becomes repulsive in water, by as much as 7 kcal/mol. The polarization energy makes up for this loss, rising from barely 1 kcal/mol in vacuum to the 8–9 kcal/mol range in water. The dispersion energy changes very little upon solvation. The analogous quantities for decomposition in Table S4 essentially mimic the aqueous results. Additional calculations performed for the **1a** dimer (in vacuum) using the SAPT protocol at the MP2/aug-cc-pVDZ level revealed that electrostatic and dispersion contributions cover 50 and 40% of total attractive interaction, respectively, in line with the LMOEDA results. 

#### 2.2.2. Acids with Negative σ-Hole

As depicted in Table 1, those Lewis acids containing either Si or Ge as tetrel atoms impart a negative charge to the σ-hole on the S or Se atom. Such a situation would argue against the formation of a chalcogen bond with a nucleophile. However, complexes do form, some in vacuum, but all within the context of solvent. The interaction energies of acids 7–18 with NCH and NCMe are collected in Table 6, which are all quite small, less than 1 kcal/mol. In the gas phase, some of these structures are only metastable, with a positive interaction energy, while others do not form at all. Interaction energies are all negative in solution, although again, only slightly so. There is a natural progression in most cases for greater stability as the environment is changed from vacuum to acetone and then to water. Figure S1 shows that AIM analysis suggests the presence of a chalcogen bond, and Tables S1–S3 confirm this supposition, again with small positive density Laplacian and energy density H. The lengths of these chalcogen bonds reported in Table 7 are rather long, some in excess of 4 Å. In addition, some are to a degree nonlinear with θ (N∙∙∙Ch-T) slightly less than 180°.

Energy decomposition reveals a fundamental distinction between these complexes and those C-containing systems with a positive σ-hole. In the first place, the electrostatic interaction is repulsive for all those listed in Table 8 in the gas phase. The polarization energy is attractive, but quite small, less than 1 kcal/mol. By far the largest contributor is the dispersion energy which accounts for roughly 80% of the attractive energy. This balance shifts dramatically when the complexes are immersed in solvent. In the first place, the electrostatic repulsion is much larger in water, as may be seen in the first column of Table 9. However, also increasing and even more so, is the polarization energy which now reaches above 20 kcal/mol. Even though solvation has little effect on the total dispersion energy, this quantity is now far outweighed by the strong polarization component so as to make up less than 6% of the total attraction. This pattern in water is essentially duplicated in acetone, as may be seen in Table S5.

## 3. Methods

Full optimizations of the complexes and the isolated monomers were performed at the MP2/aug-cc-pVDZ [28,29,30] level of theory. The pseudopotential aug-cc-pVDZ-PP basis set was used for I to account for relativistic effects [31]. This level of theory has proven its accuracy and reliability for systems of this type in numerous comparisons with larger basis sets and with various levels of treatment of electron correlation [32,33,34,35,36,37,38,39,40,41,42]. The absence of imaginary frequencies in the harmonic vibrational analyses indicated that all optimized systems represent minima on their potential energy surfaces. Calculations were performed in the gas phase, acetone (ε = 20.5) and in water (ε = 78.4), with the polarizable continuum model (PCM) [43]. The interaction energy (E_int_) of each complex was calculated as the difference between the total electronic energy of the complex and the sum of the energies of the monomers with geometries they adopt within the complex. For the gas phase, the correction of basis set superposition error (BSSE) was accounted for using the counterpoise procedure introduced by Boys and Bernardi [44]. Since the Gaussian software package does not allow the use of this method in conjunction with the PCM solvent model, the correction calculated for the solvent geometry in the gas phase was used. Calculations were performed using the Gaussian 16 software package, Rev. C.01 [45]. The decomposition of interaction energies was performed using the LMOEDA [46] scheme implemented in GAMESS-US (version 2020-R2) [47]. The MEP (molecular electrostatic potential) was measured using the MultiWFN program [48,49]. The AIM (atoms-in-molecules) method in AIMAll [50] program was used to elucidate the bond pathways and their topological properties.

## 4. Discussion

There is ample precedent for the idea that a negative MEP in the binding region of a Lewis acid molecule can nonetheless engage in a stabilizing interaction with a nucleophile. Many such examples arise in the context of a pair of interacting anions. For example, the negatively charged π-hole above the BeCl_3_^−^ anion interacts favorably with pyridine as nucleophile [51]. However, even when paired with a CN^−^ anion, the complex will form, albeit with a positive binding energy, so represents only a metastable minimum. Immersion of this anion pair in water, however, causes a strong stabilization of the complex with a clearly negative binding energy. Replacing Be by a Group 12 atom (Zn, Cd, or Hg) [12] presents a similar situation where a positive interaction energy reverses its sign when in water. It is important to stress that despite the mutual approach of a pair of anions, the electrostatic term within the complex is attractive (calculated in the gas phase for geometry taken from optimization in water using EDA implemented in ADF). Later work [52] with these spodium bonds documented a stabilizing Hg··Cl interaction between a pair of HgCl_3_^−^ anions. A more complete study which expanded the scope to other metals and halogen substituents [53] showed negative π-holes in all cases, complemented by negative interaction energies in polar solvents, despite large positive electrostatic repulsion energies (calculated in aqueous medium using LMOEDA).

A similar situation is encountered in the context of pnicogen bonds [54] involving ZCl_4_^−^, with Z = P, As, Sb, which can form between pyridine, NCH, or anionic CN^−^. Again, the electrostatic component is negative (calculated in the gas phase using EDA implemented in ADF), as is the full interaction energy, even for a pair of anions. The situation repeats itself for triel bonds to TrCl_4_^−^ anions (Tr = Al,Ga,In,Tl) [11]. The electrostatic term is negative for both neutral and anionic nucleophiles (calculated in the gas phase using ADF EDA). The full interaction energy is negative for NH_3_ but turns positive for CN^−^. However, like the situations considered here, the interaction energy becomes decidedly negative in aqueous solution. Even in the case of rare-gas atoms [55], the negative π-hole above the AeX_5_^−^ anion (Ae = Kr,Xe) interacts favorably with anions in water, but results in a positive interaction energy in less polar solvents. It is notable that the complexation process in these aerogen bonds must overcome a strongly positive LMOEDA electrostatic repulsion.

Given the importance of the numerical value of the MEP at the σ-hole in terms of the strength of any chalcogen (or other) noncovalent bond, it is important to carefully consider at precisely what point that potential ought to be evaluated. The most common prescription is to identify an isodensity surface, typically ρ = 0.001 au, and evaluate the MEP at the point where this potential achieves a maximum on this surface. The motivation for choosing this density is that it is thought to be roughly equivalent to a van der Waals surface. The distance of V_s,max_ from the chalcogen atom of each of several selected Lewis acid molecules is listed in the first column of Table 10, where it may be seen to exceed 2 Å. However, the vdW radii assigned to S and Se [56] are somewhat shorter than this, 1.89 and 1.82 Å, respectively. Moreover, in any attractive interaction, e.g., the Ch···N chalcogen bond in the gas phase, the distance between the Ch and N atoms is less than their vdW sum. In order to account for this closer approach a factor f was derived for each complex equal to the ratio between the actual R(Ch···N) distance and the sum of vdW radii. The second column of Table 10 presents the vdW radius of the Ch atom, scaled by f to account for this shrinkage. It is immediately clear that this f*vdW distance is substantially shorter than that for which the density is equal to 0.001 au in the preceding column. 

What then is the effect of evaluating the MEP at the f*vdW distance from Ch, as compared to the 0.001 au isodensity distance? The closer approach of the test point to the nucleus reduces the shielding from the surrounding electron cloud, thus making the MEP more positive. As predicted, the values in the last column of Table 10 are substantially more positive than the 0.001 au values in the preceding column. In the case of T = C Lewis acids **1–6**, this enhancement doubles or even triples the depth of the σ-hole. This closer approach is even capable of reversing the sign of V for many of the other Lewis acids in Table 1, turning a negative potential to a positive one. This alternate view of MEP might help explain how some of the acids with an ostensibly negative σ-hole are able to minimize any electrostatic repulsion and attract a nucleophile.

It is of course no surprise that the C-containing XYC=Ch molecules can engage in a chalcogen bond as there is a positively charged σ-hole in the proper position, along the C=Ch bond extension. This hole becomes even more positively charged when the reference point comes in closer to the Ch atom, more accurately located at the vdW distance. What is less obvious is that some of the other XYT=Ch molecules, with T = Si or Ge, can also engage in a stabilizing interaction with a nucleophile, despite a negative potential at the σ-hole position. However, placement of the reference point closer to the vdW radius raises the value of V_s,max_, making it positive in most cases, thereby bringing the MEP analysis into better accord with the energetics. But even so, when corrected for basis set superposition error, the interaction energies of most of these complexes become positive.

**Figure 2:**
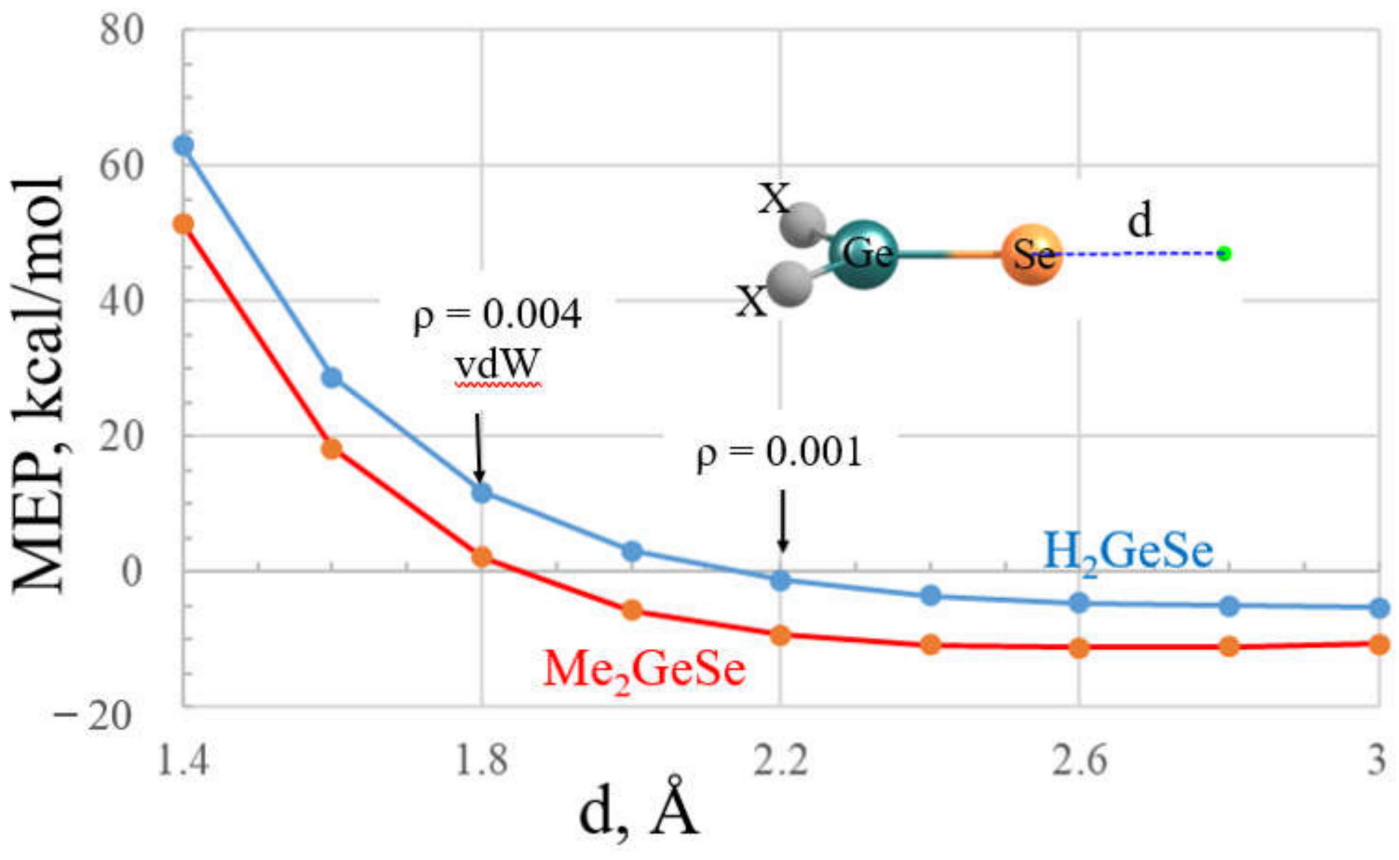
The molecular electrostatic potential at various distances d from the Se nucleus of **16,** H_2_GeSe (blue) and **18,** (CH_3_)_2_GeSe (red). Arrows indicate vdW radius of Se and the value of d for which the electron density is equal to 0.001 au.

The effects of selection of point of reference upon the MEP assigned to the σ-hole is illustrated graphically for two related molecules, H_2_GeSe (**16**) and Me_2_GeSe (**18**), in Figure 2. The MEP is shown for each molecule in terms of the distance d from the Se atom. This property rapidly declines as the point of reference moves away from the Se nucleus and is progressively better shielded by its electron cloud. The MEP begins as a positive quantity but shifts over to negative at some specific distance, which is 2.10 Å for H_2_GeSe, but even closer, at about 1.85 Å, for the dimethyl-substituted molecule. If one considers the 1.8 Å vdW radius of Se, the MEP of H_2_GeSe is clearly positive, whereas H_2_GeSe shows a value near zero. The electron density at this vdW distance is equal to 0.004 au, much larger than the 0.001 au commonly taken as the proper distance at which to assess the MEP. The density does not drop down to 0.001 au until a point that is some 2.2 Å from Se, 0.4 Å longer than the vdW radius. At this longer d, the MEP of both molecules has dropped below zero, albeit only slightly for H_2_GeSe. Given this sensitivity of the MEP to the choice of distance or isodensity surface, one should therefore exercise due caution in the manner in which the sign and magnitude of a σ-hole is interpreted.

One property that the systems described by Table 10 had in common is that each corresponds to a stable or metastable chalcogen bonded complex in the gas phase. However, there were a number of Lewis acids that engaged in such a complex only in solution, not in vacuo. It is thus of interest to consider how the MEP of each of these Lewis acids changes if one considers the vdW radius rather than the ρ = 0.001 au isodensity surface. This potential is listed in Table S6 in the gas phase as well as in solution. The change in the distance of the reference point to the vdW radius does make the potentials in Table 1 somewhat less negative, but in only one case does this quantity become positive. Moreover, immersion in solvent, whether acetone or water, makes this potential considerably more negative. The large positive electrostatic energies in Table 9 can thus be attributed in part to the Coulombic repulsion between this negatively charged σ-hole and the incoming nucleophile. It is only because of the strongly attractive polarization component that these complexes can form in solution.

Murray and Politzer [25] have recently reported a list of complexes that are formed between two neutral molecules, the acid of which contains a purportedly negative σ-hole. As evaluated on the 0.001 au isodensity surface, a V_s,max_ as large as −10 kcal/mol was nonetheless able to engage in a stable complex, albeit only weakly so. Given the sensitivity of MEP to distance from the atom in question discussed above, it is entirely possible that some of the negative σ-holes might reverse their sign to positive for closer reference points, more nearly equal to vdW radii, which would change the interpretation that these complexes are counter-intuitive. Taking H_3_SiCl as one example, the maximum on the 0.001 au isodensity surface lies some 2.03 Å from the Cl atom and has a value of −2.9 kcal/mol at the MP2/aug-cc-pVDZ level. If the MEP is evaluated instead at the vdW radius distance from the Cl atom (1.82 Å), it takes on a positive value of +1.1 kcal/mol. Unfortunately, The authors did not evaluate the full electrostatic interaction in these complexes, nor any of the other components. Nevertheless, they attributed the stability of the complexes to the effect of polarization which can in principle counteract a repulsive electrostatic component, should one be present. This idea is supported to a certain extent by the data in Table 8 in which the electrostatic and polarization energies are respectively positive and negative. However, the polarization energy is not in and of itself capable of counteracting this repulsion but must be supplemented by a large dispersion attraction. The situation changes in aqueous solution however, where the polarization component is of roughly equal magnitude to the electrostatic repulsion (see Table 9).

Focusing on the attractive elements of polarization and dispersion in the context of neutral subunits, Wang et al. [23] have calculated negative σ-holes on the halogen atoms of AuX that varied between −4.1 kcal/mol for X = I to as much as −22.2 kcal/mol for X = Cl. Despite these substantial negative values, these molecules were able to form a halogen bond of as high an interaction energy as 3.8 kcal/mol. Again, the premise of a negative σ-hole was based on evaluation at the 0.001 au isodensity surface, so might perhaps be reversed for shorter contacts, more akin to vdW radii. The electrostatic component was small and varied in sign from one X to another. The polarization energy was quite negative, but the attractive dispersion term was even larger. The importance of dispersion to permit the mutual approach of two negative areas of MEP has recently been reiterated by Zhang and Wang [24] in the context of several complexes extracted from crystal structures, where this component is much larger in magnitude than either electrostatics or polarization.

Given the sensitivity of the value of the MEP to distance from the nucleus (see Figure 2), one might be tempted to question the value of this quantity at all. Yet countless papers in the literature have demonstrated the depth of the σ or π-hole to frequently be an accurate predictor of the strength of the interaction of the Lewis acid with a partner nucleophile. It would seem that the value of this property lies in its comparison from one molecule to another. Note, for instance, the parallel nature of the curves in Figure 2. Irrespective of what distance or isodensity surface is chosen, H_2_GeSe would be predicted to have a more positive σ-hole than does Me_2_GeSe, and by a relatively constant amount. In summary, it would appear constructive to evaluate the MEP by some criterion, whether isodensity or standard distance, and then use the comparative values as a metric by which to predict relative strengths of the noncovalent bonds in which they engage.

## 5. Conclusions

Many molecules in the XYT=Ch series can engage in a chalcogen bond with the N-bases NCH and NCMe. For those Lewis acids with T = C, there is a clearly positive σ-hole along the extension of the C=Ch bond axis, which leads to an interaction energy between 1.3 and 2.4 kcal/mol. Immersion in either acetone or water weakens these chalcogen bonds a little but the interaction energy remains negative. Gas-phase interactions have only a small polarization component, with the electrostatic term slightly larger than dispersion. On the other hand, polarization plays the dominant role with solvent, where the electrostatic interaction becomes repulsive.

The sign of the σ-hole changes to negative for T = Si or Ge, as evaluated on the ρ = 0.001 au isodensity surface. Despite this, some of these Lewis acids can engage in a stable complex with the bases, albeit very weak ones. Even when immersed in solvent, these interaction energies remain below 1 kcal/mol. These dimers owe much of their stability to dispersion which must counteract a repulsive electrostatic component. Like the stronger complexes with T = C, solvation makes polarization the dominant attractive term, while amplifying the repulsive effect of the electrostatic contribution. The negative signs attributed to the σ-holes of this set of Lewis acids appear to be artifacts of the application of the ρ = 0.001 au isodensity surface, which is somewhat further from the Ch nucleus than is the vdW radius. Placing the reference point closer to the vdW distance raises the value of the MEP very substantially, such that even the negative σ-holes switch their sign.

## Figures and Tables

**Table 1:** Molecular electrostatic potential maxima (kcal/mol) on the 0.001 au isodensity surface at Ch atom of monomers (Ch = S, Se) and minima (kcal/mol) of the HCN, CH_3_CN, calculated at the MP2/aug-cc-pVDZ level of theory.

	Gas (ε = 1)^a^	R(V_s,max_) Å	Acetone (ε = 20.493)	R(V_s,max_) Å	Water (ε = 78.355)	R(V_s,max_) Å
Lewis acid	Vs,max > 0					
**1**	13.52	2.043	13.64	2.039	13.64	2.038
**2**	11.74	2.052	11.57	2.049	11.55	2.048
**3**	11.69	2.056	11.46	2.052	11.43	2.052
**4**	19.19	2.116	20.69	2.106	20.78	2.105
**5**	16.68	2.133	18.09	2.124	18.17	2.123
**6**	16.38	2.138	17.70	2.129	17.80	2.128
	Vs,max < 0					
**7**	−9.19	2.127	−19.93	2.150	−20.71	2.152
**8**	−13.67	2.135	−25.50	2.162	−26.32	2.264
**9**	−17.59	2.144	−30.57	2.173	−31.46	2.175
**10**	−1.63	2.197	−11.04	2.214	−11.70	2.215
**11**	−6.17	2.205	−16.94	2.226	−17.71	2.227
**12**	−10.14	2.213	−22.30	2.238	−23.17	2.239
**13**	−8.95	2.119	−21.68	2.146	−22.64	2.148
**14**	−13.21	2.128	−27.00	2.158	−28.01	2.160
**15**	−17.08	2.136	−31.91	2.169	−33.00	2.172
**16**	−1.08	2.189	−12.35	2.209	−13.24	2.211
**17**	−5.49	2.198	−18.03	2.222	−18.96	2.223
**18**	−9.38	2.206	−23.18	2.233	−24.22	2.236
Lewis base		R(V_s,min_) Å		R(V_s,min_) Å		R(V_s,min_) Å
HCN	−31.49	1.906	−37.29	1.931	−37.63	1.933
NCCH_3_	−37.47	1.915	−44.35	1.943	−44.75	1.945

^a^ value for the dielectric constant of the solvent.

**Table 2:** Intermolecular distances (Å) in complexes.

	R(Ch∙∙∙N)	R(Ch∙∙∙N)	R(Ch∙∙∙N)
	gas	acetone	water
**1a**	3.227	3.251	3.255
**1b**	3.194	3.231	3.239
**2a**	3.217	3.244	3.249
**2b**	3.177	3.220	3.226
**3a**	3.207	3.235	3.240
**3b**	3.165	3.210	3.216
**4a**	3.198	3.198	3.200
**4b**	3.153	3.162	3.164
**5a**	3.200	3.206	3.209
**5b**	3.149	3.166	3.171
**6a**	3.195	3.202	3.204
**6b**	3.138	3.158	3.162

**Table 3:** Interaction energies (kcal/mol) of chalcogen bonded dimers. MP2/aug-cc-pVDZ values corrected for BSSE.

	Gas	Acetone	Water
**1a**	−1.34	−0.67	−0.64
**1b**	−1.64	−0.73	−0.69
**2a**	−1.32	−0.70	−0.68
**2b**	−1.62	−0.76	−0.73
**3a**	−1.37	−0.72	−0.69
**3b**	−1.67	−0.78	−0.75
**4a**	−1.98	−1.08	−1.03
**4b**	−2.44	−1.22	−1.16
**5a**	−1.89	−1.06	−1.01
**5b**	−2.33	−1.19	−1.13
**6a**	−1.91	−1.06	−1.02
**6b**	−2.36	−1.19	−1.13

**Table 4:** LMOEDA/MP2/aug-cc-pVDZ decomposition of the interaction energy in vacuum of complexes into electrostatic (E_es_), Pauli repulsion (E_Pauli_), polarization (E_pol_) and dispersion (E_disp_) components. All quantities in kcal/mol.

	E_es_	%^A^	E_Pauli_^B^	E_pol_	%	E_disp_	%	E_int_
**1a**	−1.66	48	2.04	−0.50	14	−1.29	37	−1.41
**1b**	−2.03	50	2.37	−0.69	17	−1.38	34	−1.73
**2a**	−1.41	39	2.19	−0.56	16	−1.61	45	−1.39
**2b**	−1.73	40	2.61	−0.79	18	−1.79	42	−1.70
**3a**	−1.46	39	2.29	−0.59	16	−1.65	45	−1.42
**3b**	−1.81	40	2.74	−0.85	19	−1.83	41	−1.74
**4a**	−2.60	52	2.97	−0.75	15	−1.62	33	−2.01
**4b**	−3.25	54	3.59	−1.04	17	−1.77	29	−2.47
**5a**	−2.27	45	3.10	−0.80	16	−1.95	39	−1.92
**5b**	−2.86	46	3.81	−1.13	18	−2.19	35	−2.37
**6a**	−2.31	45	3.19	−0.84	16	−1.97	38	−1.93
**6b**	−2.95	46	4.01	−1.21	19	−2.23	35	−2.38

^A^ percentage contribution to total attractive interactions (E_es_ + E_pol_ + E_disp_), ^B^ E_Pauli_ = ∑(E_ex_ + E_rep_).

**Table 5:** LMOEDA/MP2/aug-cc-pVDZ decomposition of the interaction energy in water of complexes into electrostatic (E_es_), Pauli repulsion (E_Pauli_), polarization (E_pol_) and dispersion (E_disp_) components. All quantities in kcal/mol.

	E_es_	E_Pauli_^A^	E_pol_	%^B^	E_disp_	%	E_int_
**1a**	5.94	1.90	−7.68	86	−1.21	14	−1.05
**1b**	6.51	2.09	−8.52	87	−1.29	13	−1.20
**2a**	6.54	2.01	−8.20	85	−1.43	15	−1.07
**2b**	7.20	2.26	−9.15	86	−1.55	14	−1.23
**3a**	6.65	2.09	−8.36	85	−1.48	15	−1.09
**3b**	7.30	2.37	−9.31	85	−1.61	15	−1.25
**4a**	4.72	3.02	−7.74	83	−1.53	17	−1.53
**4b**	4.90	3.54	−8.56	84	−1.67	16	−1.79
**5a**	5.45	3.08	−8.26	82	−1.76	18	−1.49
**5b**	5.77	3.63	−9.19	82	−1.95	18	−1.74
**6a**	5.20	3.17	−8.05	82	−1.82	18	−1.51
**6b**	5.87	3.79	−9.38	82	−2.02	18	−1.74

^A^ E_Pauli_ = ∑(E_ex_ + E_rep_), ^B^ percentage contribution to total attractive interactions (E_pol_ + E_disp_).

**Table 6:** Interaction energies (kcal/mol) of chalcogen bonded dimers calculated at the MP2/aug-cc-pVDZ. Values corrected for BSSE.

	Gas	Acetone	Water
**7a**	0.40	−0.15	−0.25
**7b**	-^A^	−0.15	−0.27
**8a**	-	−0.13	−0.26
**8b**	-	−0.12	−0.27
**9a**	-	−0.10	−0.25
**9b**	-	−0.09	−0.26
**10a**	−0.17	−0.16	−0.22
**10b**	−0.19	−0.14	−0.22
**11a**	0.19	−0.09	−0.17
**11b**	0.27	−0.06	−0.16
**12a**	0.51	−0.02	−0.13
**12b**	0.66	0.03	−0.11
**13a**	0.37	−0.11	−0.24
**13b**	-	−0.14	−0.27
**14a**	-	−0.11	−0.26
**14b**	-	−0.12	−0.28
**15a**	-	−0.11	−0.26
**15b**	1.10	−0.09	−0.27
**16a**	−0.24	−0.18	−0.24
**16b**	−0.27	−0.15	−0.24
**17a**	0.12	−0.11	−0.20
**17b**	0.17	−0.08	−0.20
**18a**	0.43	−0.05	−0.16
**18b**	0.57	−0.01	−0.25

^A^ there are no stable minima with chalcogen bond.

**Table 7:** Geometrical parameters (distances in Å, angles in deg) for complexes calculated at the MP2/aug-cc-pVDZ level of theory.

	R(Ch∙∙∙N)	θ (N∙∙∙Ch-T)	R(Ch∙∙∙N)	θ(N∙∙∙Ch-T)	R(Ch∙∙∙N)	θ(N∙∙∙Ch-T)
	gas		acetone		water	
**7a**	3.540	180.0	4.070	172.7	4.069	173.1
**7b**	-^A^	-	4.127	172.5	4.126	172.8
**8a**	-	-	4.123	176.9	4.123	177.1
**8b**	-	-	4.141	177.7	4.139	177.6
**9a**	-	-	4.146	176.2	4.142	175.5
**9b**	-	-	4.148	176.7	4.158	174.1
**10a**	3.451	180.0	3.632	179.1	3.652	179.2
**10b**	3.416	179.8	3.634	179.4	3.659	179.5
**11a**	3.487	179.0	3.684	180.0	3.701	179.9
**11b**	3.457	179.5	3.694	179.8	3.709	179.7
**12a**	3.521	180.0	3.724	179.4	3.748	179.5
**12b**	3.499	180.0	3.727	179.7	3.748	179.5
**13a**	3.461	180.0	3.884	178.1	3.945	175.8
**13b**	-	-	3.992	179.2	3.994	178.7
**14a**	-	-	4.026	175.4	4.092	175.4
**14b**	-	-	4.108	176.2	4.122	175.7
**15a**	-	-	4.128	178.8	4.124	179.0
**15b**	4.098	180.0	4.135	179.3	4.143	179.1
**16a**	3.390	180.0	3.572	179.3	3.589	179.1
**16b**	3.356	179.9	3.568	179.5	3.595	179.2
**17a**	3.430	179.5	3.641	179.5	3.662	179.3
**17b**	3.395	180.0	3.645	179.5	3.677	179.5
**18a**	3.462	180.0	3.691	179.1	3.709	179.2
**18b**	3.432	179.9	3.697	179.1	3.715	179.3

^A^ there are no stable minima with chalcogen bond.

**Table 8:** LMOEDA/MP2/aug-cc-pVDZ decomposition of the interaction energy in vacuum of complexes into electrostatic (E_es_), Pauli repulsion (E_Pauli_), polarization (E_pol_) and dispersion (E_disp_) components. All quantities in kcal/mol.

	E_es_	E_Pauli_^A^	E_pol_	%^B^	E_disp_	%	E_int_
**7a**	1.17	0.94	−0.29	17	−1.45	83	0.37
**10a**	0.33	1.74	−0.43	19	−1.82	81	−0.18
**10b**	0.51	2.00	−0.61	23	−2.10	77	−0.20
**11a**	0.81	1.57	−0.41	19	−1.79	81	0.18
**11b**	1.11	1.78	−0.58	22	−2.06	78	0.25
**12a**	1.22	1.44	−0.40	19	−1.75	81	0.50
**12b**	1.63	1.58	−0.56	22	−2.02	78	0.64
**13a**	1.18	1.19	−0.35	18	−1.64	82	0.38
**15b**	2.26	0.15	−0.23	18	−1.08	82	1.10
**16a**	0.23	2.05	−0.51	20	−1.99	80	−0.22
**16b**	0.39	2.35	−0.72	24	−2.28	76	−0.25
**17a**	0.73	1.84	−0.48	20	−1.96	80	0.14
**17b**	1.02	2.12	−0.68	23	−2.27	77	0.19
**18a**	1.16	1.69	−0.47	20	−1.93	80	0.45
**18b**	1.57	1.91	−0.66	23	−2.24	77	0.58

^A^ E_Pauli_ = ∑(E_ex_ + E_rep_), ^B^ percentage contribution to total attractive interactions (E_pol_ + E_disp_).

**Table 9:** LMOEDA/MP2/aug-cc-pVDZ decomposition of the interaction energy in water of complexes into electrostatic (E_es_), repulsion (E_rep_), exchange (E_ex_), polarization (E_pol_) and dispersion (E_disp_) components. All quantities in kcal/mol.

	E_es_	E_Pauli_^A^	E_pol_	%^B^	E_disp_	%	E_int_
water							
**7a**	18.85	0.16	−18.77	97	−0.53	3	−0.28
**7b**	20.44	0.13	−20.34	97	−0.53	3	−0.30
**8a**	20.58	0.14	−20.48	98	−0.50	2	−0.27
**8b**	22.28	0.13	−22.17	98	−0.53	2	−0.29
**9a**	22.30	0.13	−22.20	98	−0.50	2	−0.26
**9b**	24.11	0.13	−23.99	98	−0.53	2	−0.28
**10a**	17.30	0.91	−17.48	94	−1.13	6	−0.41
**10b**	18.83	0.91	−18.99	94	−1.20	6	−0.46
**11a**	19.33	0.79	−19.39	95	−1.07	5	−0.33
**11b**	21.00	0.79	−21.03	95	−1.13	5	−0.37
**12a**	21.30	0.7	−21.27	95	−1.01	5	−0.28
**12b**	23.11	0.71	−23.03	95	−1.09	5	−0.31
**13a**	21.59	0.24	−21.48	97	−0.65	3	−0.30
**13b**	23.25	0.2	−23.13	97	−0.66	3	−0.33
**14a**	23.28	0.15	−23.17	98	−0.54	2	−0.29
**14b**	25.06	0.14	−24.94	98	−0.57	2	−0.31
**15a**	25.00	0.14	−24.89	98	−0.53	2	−0.28
**15b**	26.94	0.13	−26.80	98	−0.56	2	−0.29
**16a**	19.57	1.08	−19.84	94	−1.25	6	−0.43
**16b**	21.07	1.09	−21.32	94	−1.33	6	−0.50
**17a**	21.64	0.88	−21.73	95	−1.15	5	−0.37
**17b**	23.39	0.85	−23.44	95	−1.21	5	−0.42
**18a**	23.62	0.77	−23.61	96	−1.10	4	−0.31
**18b**	25.49	0.78	−25.43	96	−1.17	4	−0.34

^A^ E_Pauli_ = ∑(E_ex_ + E_rep_), ^B^ percentage contribution to total attractive interactions (E_pol_ + E_disp_).

**Table 10:** Distances of points for evaluating MEP from Ch atom, and value of MEP at that point.

	d, Å		V, kcal/mol	
	ρ = 0.001	f*vdW	ρ = 0.001	f*vdW
**1a**	2.043	1.718	13.52	27.49
**1b**		1.700		28.73
**2a**	2.052	1.713	11.74	26.04
**2b**		1.691		27.58
**3a**	2.056	1.707	11.69	26.49
**3b**		1.685		28.05
**4a**	2.116	1.673	19.19	46.03
**4b**		1.649		48.67
**5a**	2.133	1.674	16.68	43.48
**5b**		1.647		46.43
**6a**	2.138	1.671	16.38	43.62
**6b**		1.641		46.94
**10a**	2.197	1.805	−1.63	10.47
**10b**		1.787		11.51
**11a**	2.205	1.824	−6.17	4.11
**11b**		1.841		3.29
**12a**	2.213	1.841	−10.14	−1.37
**12b**		1.830		−0.87
**16a**	2.189	1.773	−1.08	13.45
**16b**		1.755		14.66
**17a**	2.198	1.794	−5.49	7.01
**17b**		1.776		8.09
**18a**	2.206	1.811	−9.38	1.48
**18b**		1.795		2.35

## Data Availability

The data presented in this study are available in this article or as Supplementary Materials.

## Supplementary Materials

Figure S1: AIM molecular diagrams of MP2/aug-cc-pVDZ optimized complexes. Green dots represent bond critical points. Numbers refers to electron densities at BCPs (in au), Table S1: AIM descriptors of the calculated complexes in vacuum. Bond critical point (BCP) properties: electron density *ρ*, Laplacian of electron density ∇^2^*ρ* and total electron energy H and potential electron density energy V as well as kinetic electron density energy G, were obtained at the MP2/ aug-cc-pVDZ level. Data in atomic units, Table S2: AIM descriptors of the calculated complexes in acetone. Bond critical point (BCP) properties: electron density *ρ*, Laplacian of electron density ∇^2^*ρ* and total electron energy H and potential electron density energy V as well as kinetic electron density energy G, were obtained at the MP2/ aug-cc-pVDZ level. Data in atomic units, Table S3: AIM descriptors of the calculated complexes in water. Bond critical point (BCP) properties: electron density *ρ*, Laplacian of electron density ∇^2^*ρ* and total electron energy H and potential electron density energy V as well as kinetic electron density energy G, were obtained at the MP2/ aug-cc-pVDZ level. Data in atomic units, Table S4: LMOEDA/MP2/aug-cc-pVDZ decomposition of the interaction energy in acetone of complexes into electrostatic (E_es_), Pauli repulsion (E_Pauli_), polarization (E_pol_) and dispersion (E_disp_) components. All quantities in kcal/mol, Table S5: LMOEDA/MP2/aug-cc-pVDZ decomposition of the interaction energy in acetone of complexes into electrostatic (E_es_), Pauli repulsion (E_Pauli_), polarization (E_pol_) and dispersion (E_disp_) components. All quantities in kcal/mol, Table S6: MEP (kcal/mol of each monomer at a point along the extension of the T=Ch bond axis, at a distance from Ch that corresponds to its vdW radius in three different phases, Table S7: Atomic polar tensor (APT) charges (e) on chalcogen atom in monomers in vacuum, acetone and water solvents. Calculations performed at the MP2/aug-cc-pVDZ level of theory, Table S8: Coordinates of monomers, Table S9: Coordinates of dimers.

## Sample Availability

Samples of the compounds are not available from the authors.

## References

[1] Zhang Y., Wang W., Wang Y.-B. (2019). Tetrel bonding on graphene. Comput. Theor. Chem..

[2] Esrafili M.D., Mousavian P. (2018). Strong Tetrel Bonds: Theoretical Aspects and Experimental Evidence. Molecules.

[3] Esrafili M.D., Asadollahi S., Mousavian P. (2018). Anionic tetrel bonds: An ab initio study. Chem. Phys. Lett..

[4] Wu J., Yan H., Zhong A., Chen H., Jin Y., Dai G. (2019). Theoretical and conceptual DFT study of pnicogen- and halogen-bonded complexes of PH2X---BrCl. J. Mol. Model..

[5] Guan L., Mo Y. (2014). Electron transfer in pnicogen bonds. J. Phys. Chem. A.

[6] Ben Aissa M.A., Hassen S., Arfaoui Y. (2019). Theoretical Density Functional Theory insights into the nature of chalcogen bonding between CX 2 (X = S, Se, Te) and diazine from monomer to supramolecular complexes. Int. J. Quantum Chem..

[7] Pascoe D.J., Ling K.B., Cockroft S.L. (2017). The Origin of Chalcogen-Bonding Interactions. J. Am. Chem. Soc..

[8] Stone A.J. (2013). Are halogen bonded structures electrostatically driven?. J. Am. Chem. Soc..

[9] Orlova A.P., Jasien P.G. (2018). Halogen bonding in self-assembling systems: A comparison of intra- and interchain binding energies. Comput. Theor. Chem..

[10] Sulaiman A.A., Zierkiewicz W., Michalczyk M., Malik-Gajewska M., Ahmad S., Alhoshani A., Sobeai H.M.A., Bienko D., Isab A.A. (2020). Synthesis, characterization, DFT optimization and anticancer evaluation of phosphanegold(I) dithiocarbamates. J. Mol. Struct..

[11] Wysokinski R., Michalczyk M., Zierkiewicz W., Scheiner S. (2021). Anion-anion and anion-neutral triel bonds. Phys. Chem. Chem. Phys..

[12] Wysokinski R., Zierkiewicz W., Michalczyk M., Scheiner S. (2020). Anion center dot center dot center dot Anion Attraction in Complexes of MCl3- (M=Zn, Cd, Hg) with CN-. Chemphyschem.

[13] Arunan E., Desiraju G.R., Klein R.A., Sadlej J., Scheiner S., Alkorta I., Clary D.C., Crabtree R.H., Dannenberg J.J., Hobza P. (2011). Definition of the hydrogen bond (IUPAC Recommendations 2011). Pure Appl. Chem..

[14] Desiraju G.R., Ho P.S., Kloo L., Legon A.C., Marquardt R., Metrangolo P., Politzer P., Resnati G., Rissanen K. (2013). Definition of the halogen bond (IUPAC Recommendations 2013). Pure Appl. Chem..

[15] Aakeroy C.B., Bryce D.L., Desiraju G.R., Frontera A., Legon A.C., Nicotra F., Rissanen K., Scheiner S., Terraneo G., Metrangolo P. (2019). Definition of the chalcogen bond (IUPAC Recommendations 2019). Pure Appl. Chem..

[16] Murray J.S., Lane P., Clark T., Riley K.E., Politzer P. (2012). Sigma-holes, pi-holes and electrostatically-driven interactions. J. Mol. Model..

[17] Murray J.S., Lane P., Politzer P. (2009). Expansion of the sigma-hole concept. J. Mol. Model..

[18] Politzer P., Murray J.S., Concha M.C. (2008). Sigma-hole bonding between like atoms; a fallacy of atomic charges. J. Mol. Model..

[19] Murray J.S., Lane P., Clark T., Politzer P. (2007). Sigma-hole bonding: Molecules containing group VI atoms. J. Mol. Model..

[20] Clark T., Hennemann M., Murray J.S., Politzer P. (2007). Halogen bonding: The sigma-hole. Proceedings of “Modeling interactions in biomolecules II”, Prague, September 5th–9th, 2005. J. Mol. Model..

[21] Bauza A., Mooibroek T.J., Frontera A. (2015). Directionality of pi-holes in nitro compounds. Chem. Commun. Camb..

[22] Bauza A., Mooibroek T.J., Frontera A. (2015). The bright future of unconventional sigma/pi-hole interactions. Chemphyschem.

[23] Wang R.J., Li Q.Z., Scheiner S. (2020). Complexes of HArF and AuX (X = F, Cl, Br, I). Comparison of H-bonds, halogen bonds, F-shared bonds and covalent bonds. Appl. Organomet. Chem..

[24] Zhang Y., Wang W.Z. (2020). The sigma-hole sigma-hole stacking interaction: An unrecognized type of noncovalent interaction. J. Chem. Phys..

[25] Murray J.S., Politzer P. (2021). Can Counter-Intuitive Halogen Bonding Be Coulombic?. Chemphyschem.

[26] Iwamoto T., Sato K., Ishida S., Kabuto C., Kira M. (2006). Synthesis, Properties, and Reactions of a Series of Stable Dialkyl-Substituted Silicon−Chalcogen Doubly Bonded Compounds. J. Am. Chem. Soc..

[27] Matsumoto T., Tokitoh N., Okazaki R. (1999). The First Kinetically Stabilized Germanethiones and Germaneselones: Syntheses, Structures, and Reactivities. J. Am. Chem. Soc..

[28] Moller C., Plesset M.S. (1934). Note on an approximation treatment for many-electron systems. Phys. Rev..

[29] Dunning T.H. (1989). Gaussian-Basis Sets for Use in Correlated Molecular Calculations. 1. The Atoms Boron through Neon and Hydrogen. J. Chem. Phys..

[30] Woon D.E., Dunning T.H. (1993). Gaussian-Basis Sets for Use in Correlated Molecular Calculations. 3. The Atoms Aluminum through Argon. J. Chem. Phys..

[31] Peterson K.A., Figgen D., Goll E., Stoll H., Dolg M. (2003). Systematically convergent basis sets with relativistic pseudopotentials. II. Small-core pseudopotentials and correlation consistent basis sets for the post-d group 16-18 elements. J. Chem. Phys..

[32] Costa P.J. (2017). The halogen bond: Nature and applications. Phys. Sci. Rev..

[33] Devore D.P., Ellington T.L., Shuford K.L. (2020). Interrogating the Interplay between Hydrogen and Halogen Bonding in Graphitic Carbon Nitride Building Blocks. J. Phys. Chem. A.

[34] Hong Y.M., Lu Y.X., Zhu Z.D., Xu Z.J., Liu H.L. (2020). Metalloids as halogen bond acceptors: A combined crystallographic data and theoretical investigation. Chem. Phys. Lett..

[35] Saberinasab M., Salehzadeh S., Solimannejad M. (2016). The effect of a strong cation center dot center dot center dot pi interaction on a weak selenium center dot center dot center dot pi interaction: A theoretical study. Comput. Theor. Chem..

[36] Scheiner S. (2021). Comparison of Bifurcated Halogen with Hydrogen Bonds. Molecules.

[37] Scheiner S. (2021). Carbon as an electron donor atom. Polyhedron.

[38] Scheiner S. (2021). Origins and properties of the tetrel bond. Phys. Chem. Chem. Phys..

[39] Spada L., Gou Q., Geboes Y., Herrebout W.A., Melandri S., Caminati W. (2016). Rotational Study of Dimethyl Ether-Chlorotrifluoroethylene: Lone Pair center dot center dot center dot pi Interaction Links the Two Subunits. J. Phys. Chem. A.

[40] Tondro T., Roohi H. (2020). Substituent effects on the halogen and pnictogen bonds characteristics in ternary complexes 4-YPhNH2 center dot center dot center dot PH2F center dot center dot center dot ClX (Y = H, F, CN, CHO, NH2, CH3, NO2 and OCH3, and X = F, OH, CN, NC, FCC and NO2): A theoretical study. J. Chem. Sci..

[41] Yang J.M., Yu Q.W., Yang F.L., Lu K., Yan C.X., Dou W., Yang L.Z., Zhou P.P. (2020). Competition and cooperativity of hydrogen-bonding and tetrel-bonding interactions involving triethylene diamine (DABCO), H2O and CO2 in air. New J. Chem..

[42] Zhao Q. (2020). Mutual influence of tetrel and halogen bonds between XCN (X=Cl, Br) and 4-TF3-pyridine (T=C, Si, Ge). J. Mol. Model..

[43] Tomasi J., Mennucci B., Cammi R. (2005). Quantum mechanical continuum solvation models. Chem. Rev..

[44] Boys S.F., Bernardi F. (1970). Calculation of Small Molecular Interactions by Differences of Separate Total Energies-Some Procedures with Reduced Errors. Mol. Phys..

[45] Frisch M.J., Trucks G.W., Schlegel H.B., Scuseria G.E., Robb M.A., Cheeseman J.R., Scalmani G., Barone V., Petersson G.A., Nakatsuji H. Gaussian 16 Rev. C.01; Wallingford, CT, USA, 2016.

[46] Su P.F., Li H. (2009). Energy decomposition analysis of covalent bonds and intermolecular interactions. J. Chem. Phys..

[47] Barca G.M.J., Bertoni C., Carrington L., Datta D., De Silva N., Deustua J.E., Fedorov D.G., Gour J.R., Gunina A.O., Guidez E. (2020). Recent developments in the general atomic and molecular electronic structure system. J. Chem. Phys..

[48] Lu T., Chen F.W. (2012). Multiwfn: A multifunctional wavefunction analyzer. J. Comput. Chem..

[49] Lu Y.X., Liu Y.T., Xu Z.J., Li H.Y., Liu H.L., Zhu W.L. (2012). Halogen bonding for rational drug design and new drug discovery. Expert Opin. Drug Dis..

[50] Keith T.A. (2014). AIMAll.

[51] Zierkiewicz W., Wysokiński R., Michalczyk M., Scheiner S. (2020). On the Stability of Interactions between Pairs of Anions–Complexes of MCl_3_^−^ (M=Be, Mg, Ca, Sr, Ba) with Pyridine and CN^−^. Chem. Phys. Chem..

[52] Wysokiński R., Zierkiewicz W., Michalczyk M., Scheiner S. (2021). Crystallographic and Theoretical Evidences of Anion⋅⋅⋅Anion Interaction. Chem. Phys. Chem..

[53] Wysokiński R., Zierkiewicz W., Michalczyk M., Scheiner S. (2021). Anion⋯anion (MX3−)2 dimers (M = Zn, Cd, Hg; X = Cl, Br, I) in different environments. Phys. Chem. Chem. Phys..

[54] Scheiner S., Wysokiński R., Michalczyk M., Zierkiewicz W. (2020). Pnicogen Bonds Pairing Anionic Lewis Acid with Neutral and Anionic Bases. J. Phys. Chem. A.

[55] Grabarz A., Michalczyk M., Zierkiewicz W., Scheiner S. (2021). Anion–Anion Interactions in Aerogen-Bonded Complexes. Influence of Solvent Environment. Molecules.

[56] Alvarez S. (2013). A cartography of the van der Waals territories. Dalton Trans..

