# Supplementary material for: Ability of Lewis Acids with Shallow σ-Holes to Engage in Chalcogen Bonds in Different Environments
Source: Molecules. 2021 Oct 22;26(21):6394. doi: 10.3390/molecules26216394 (PMC8586936; doi:10.3390/molecules26216394)
Supplement: Supplementary file 1 [file molecules-26-06394-s001.zip › molecules-1414302-supplementary.pdf]

SUPPLEMENTARY INFORMATION

Ability of Lewis Acids with Shallow σ-Holes to Engage in Chalcogen Bonds in Different Environments

Rafał Wysokiński,*[1] Wiktor Zierkiewicz, *[1] Mariusz Michalczyk,[1] and Steve Scheiner*[2]

<table>
<tr><th></th><th>gas</th><th>acetone</th><th>water</th></tr>
<tr><td>1a</td>
<td>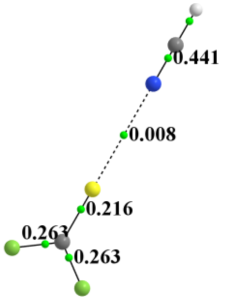</td>
<td>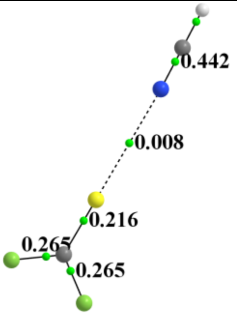</td>
<td>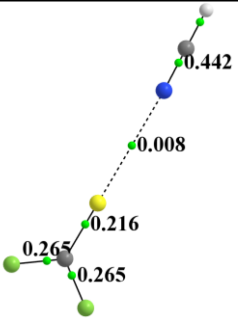</td></tr>
<tr><td>1b</td>
<td>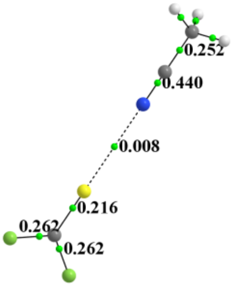</td>
<td>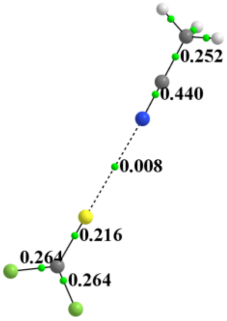</td>
<td>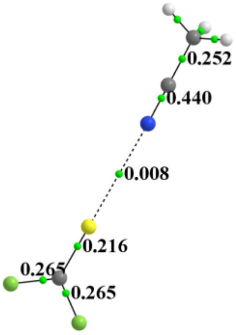</td></tr>
<tr><td>2a</td>
<td>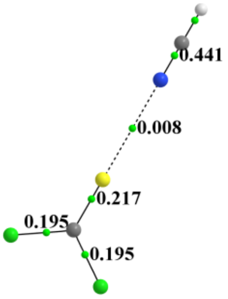</td>
<td>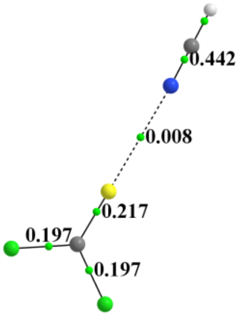</td>
<td>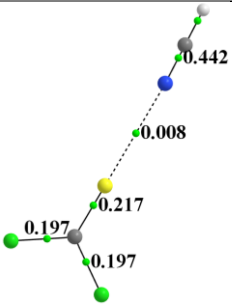</td></tr>
<tr><td>2b</td>
<td>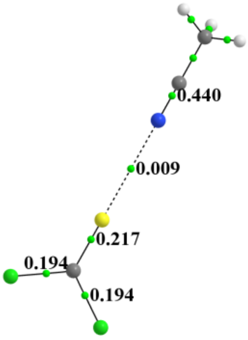</td>
<td>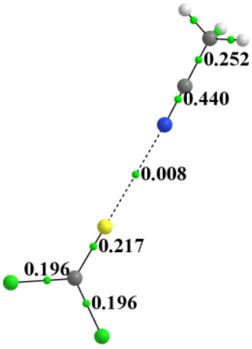</td>
<td>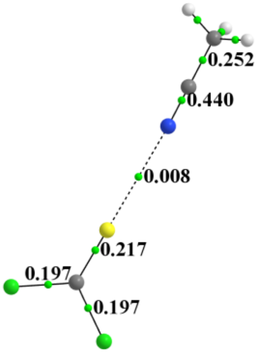</td></tr>
</table>

<table>
<tr><td>3a</td>
<td>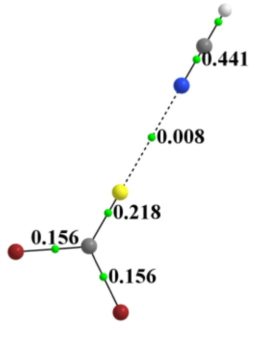</td>
<td>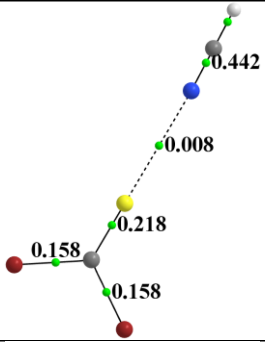</td>
<td>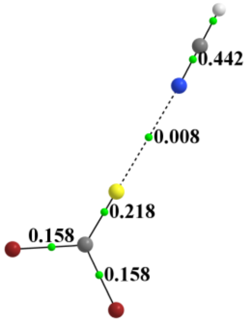</td></tr>
<tr><td>3b</td>
<td>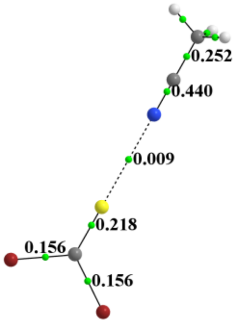</td>
<td>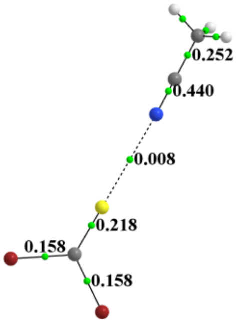</td>
<td>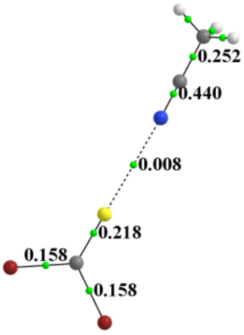</td></tr>
<tr><td>4a</td>
<td>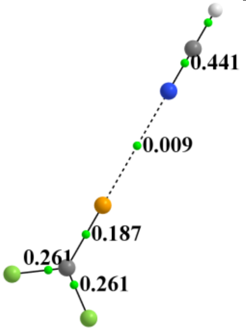</td>
<td>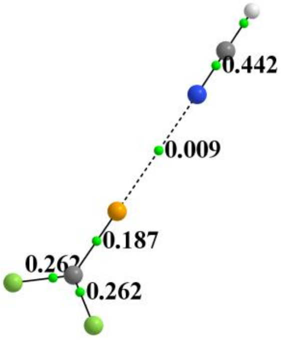</td>
<td>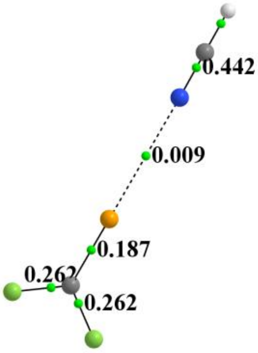</td></tr>
<tr><td>4b</td>
<td>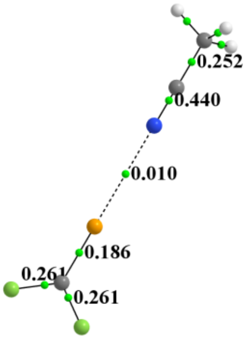</td>
<td>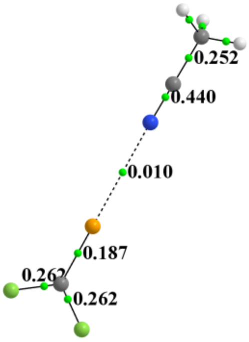</td>
<td>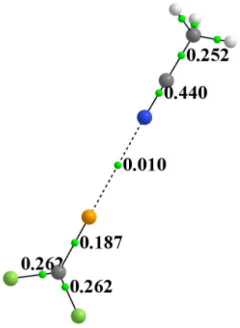</td></tr>
<tr><td>5a</td>
<td>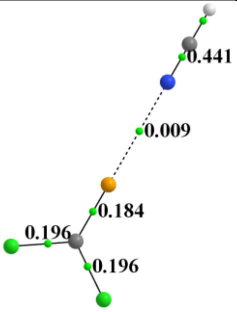</td>
<td>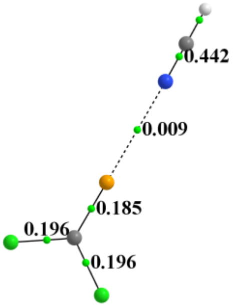</td>
<td>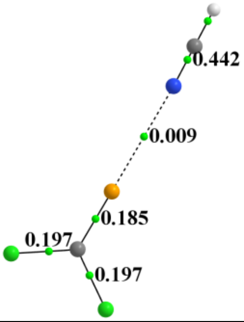</td></tr>
</table>

<table>
<tr><td>5b</td><td>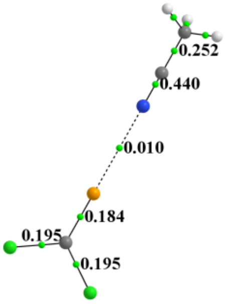
</td><td>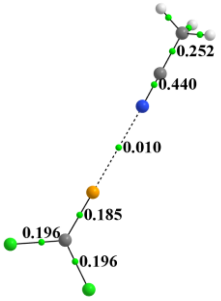
</td><td>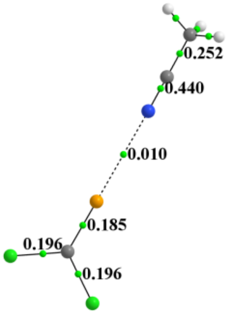
</td></tr>
<tr><td>6a</td><td>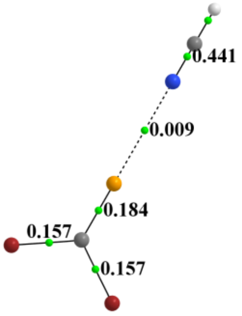
</td><td>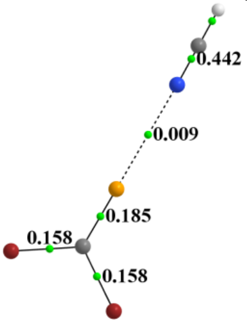
</td><td>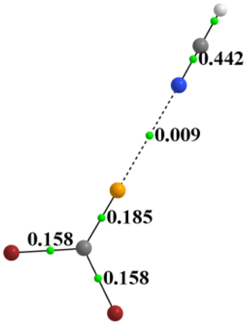
</td></tr>
<tr><td>6b</td><td>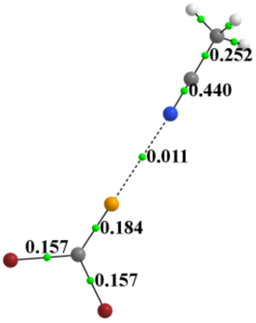
</td><td>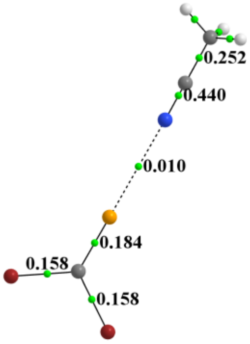
</td><td>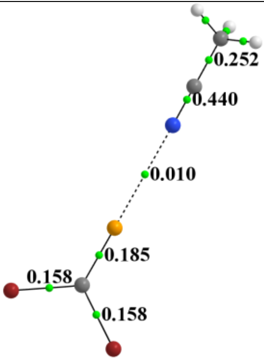
</td></tr>
<tr><td></td><td></td><td></td><td></td></tr>
<tr><td>7a</td><td>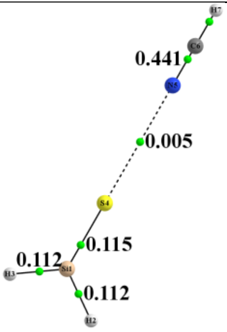
</td><td>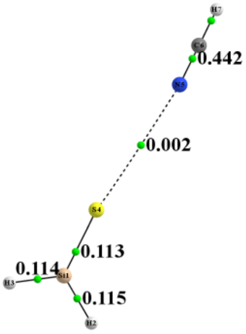
</td><td>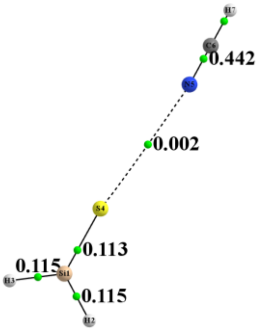
</td></tr>
<tr><td>7b</td><td></td><td>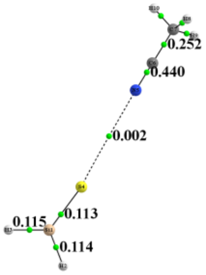
</td><td>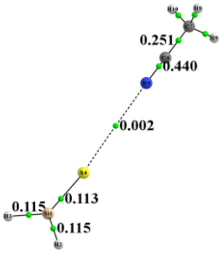
</td></tr>
</table>

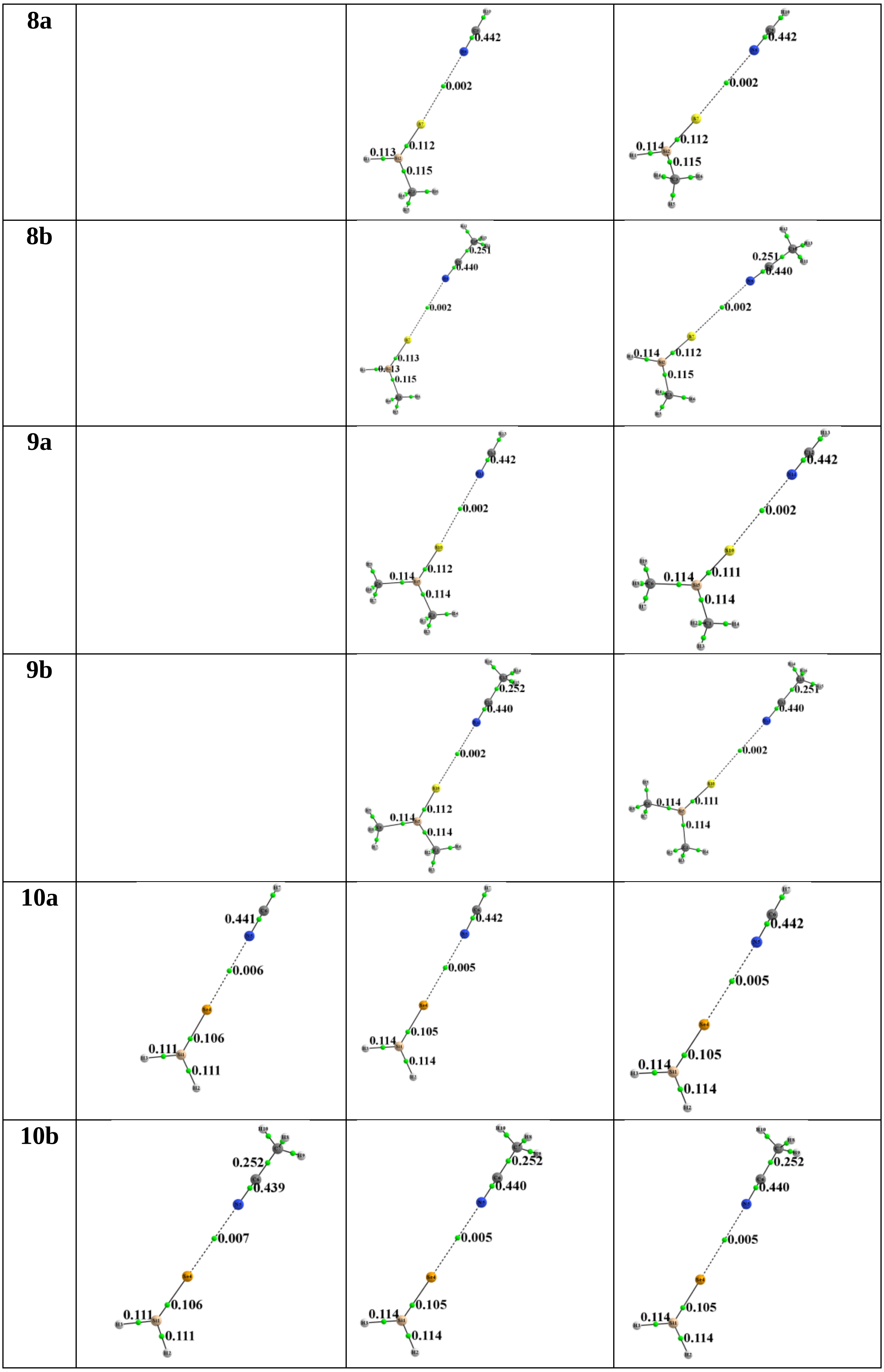

8a
0.442
0.002
0.113
0.112
0.115
0.442
0.002
0.114
0.112
0.115
8b
0.251
0.440
0.002
0.113
0.115
0.251
0.440
0.002
0.114
0.112
0.115
9a
0.442
0.002
0.114
0.112
0.114
0.442
0.002
0.114
0.111
0.114
9b
0.252
0.440
0.002
0.114
0.112
0.114
0.251
0.440
0.002
0.114
0.111
0.114
10a
0.441
0.006
0.111
0.106
0.111
0.442
0.005
0.114
0.105
0.114
0.442
0.005
0.114
0.105
0.114
10b
0.252
0.439
0.007
0.111
0.106
0.111
0.252
0.440
0.005
0.114
0.105
0.114
0.252
0.440
0.005
0.114
0.105
0.114

**11a**	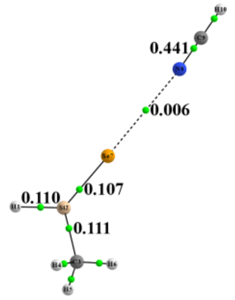	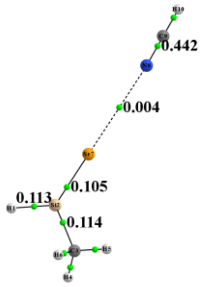	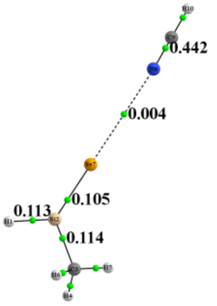
**11b**	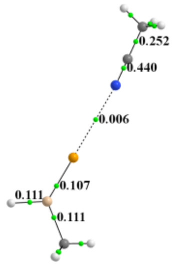	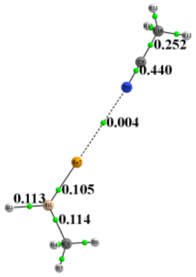	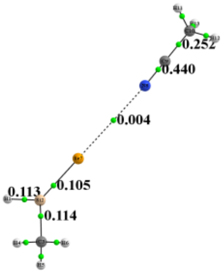
**12a**	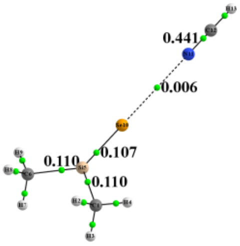	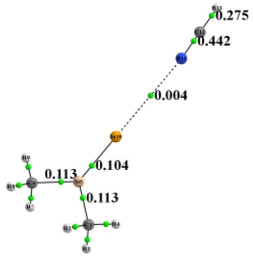	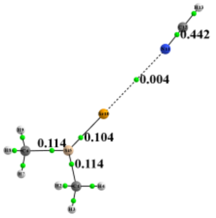
**12b**	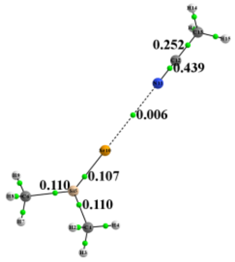	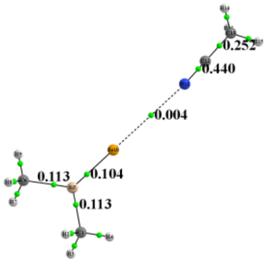	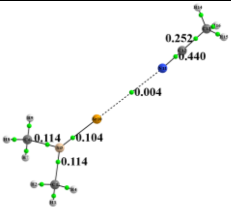
**13a**	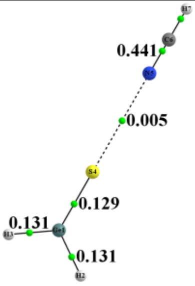	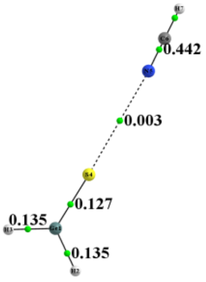	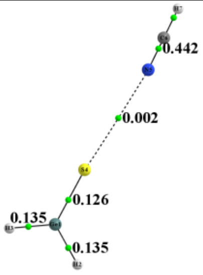
**13b**		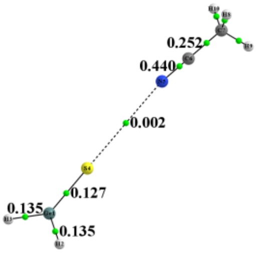	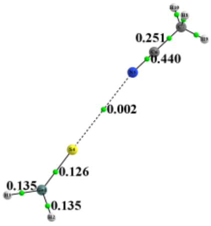

<table>
<tr><td>14a</td><td></td><td>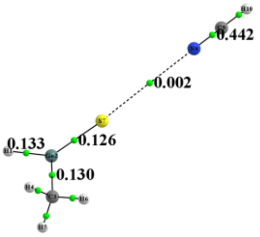
</td><td>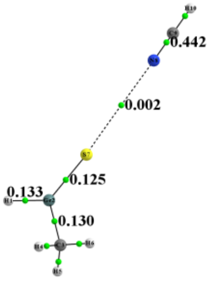
</td></tr>
<tr><td>14b</td><td></td><td>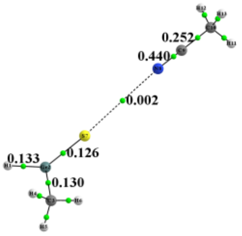
</td><td>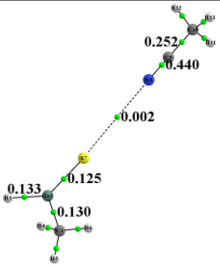
</td></tr>
<tr><td>15a</td><td></td><td>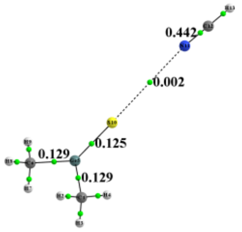
</td><td>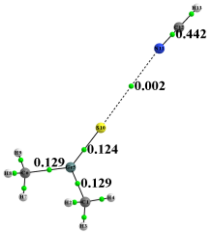
</td></tr>
<tr><td>15b</td><td>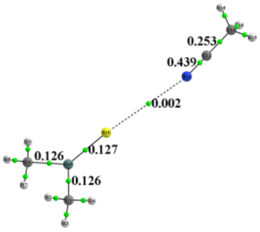
</td><td>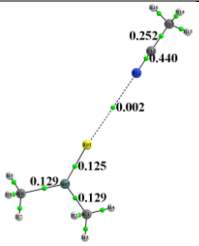
</td><td>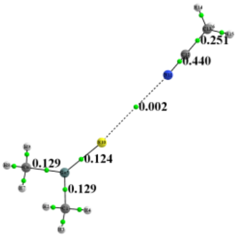
</td></tr>
<tr><td>16a</td><td>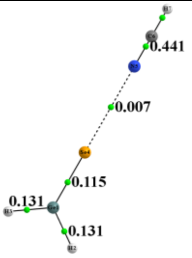
</td><td>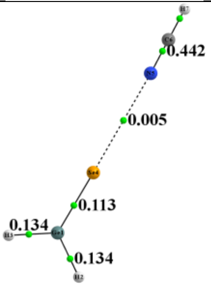
</td><td>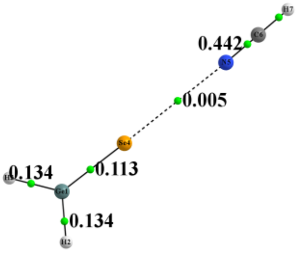
</td></tr>
<tr><td>16b</td><td>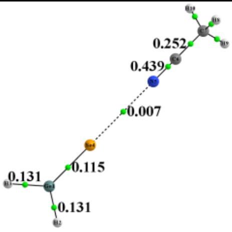
</td><td>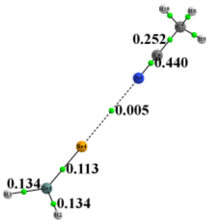
</td><td>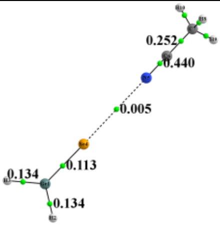
</td></tr>
</table>

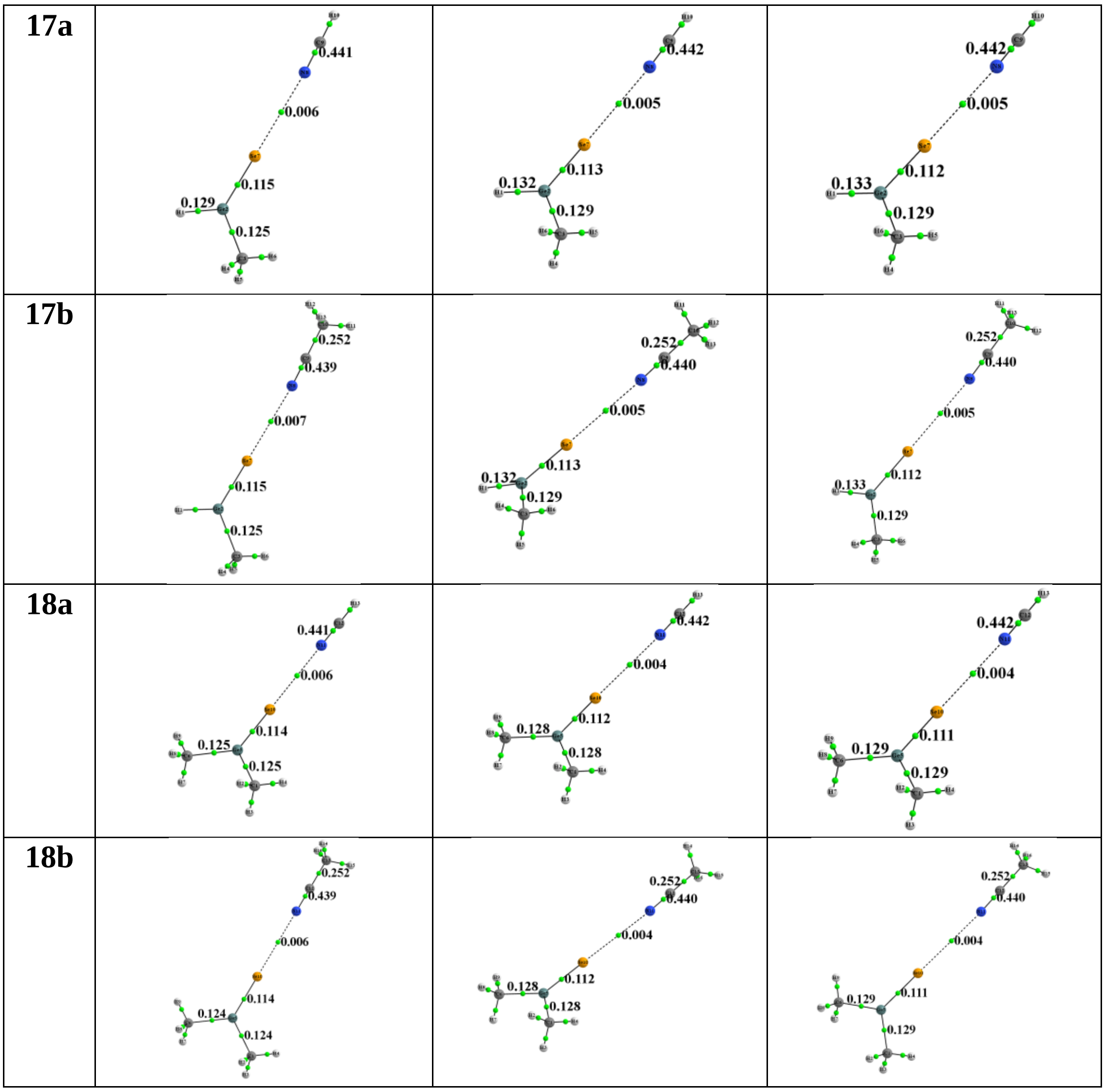

Fig. S1. AIM molecular diagrams of MP2/aug-cc-pVDZ optimized complexes. Green dots represent bond critical points. Numbers refers to electron densities at BCPs (in au)

Table S1. AIM descriptors of the calculated complexes in vacuum. Bond critical point (BCP) properties: electron density ρ, Laplacian of electron density $\nabla^2\rho$ and total electron energy H and potential electron density energy V as well as kinetic electron density energy G, were obtained at the MP2/ aug-cc-pVDZ level. Data in atomic units.

	interaction	ρ	$\nabla^2\rho$	H	V	G
**1a**	S···N	0.008	+0.026	+0.001	-0.004	+0.005
	C-S	0.216	+0.506	-0.224	-0.575	+0.351
**1b**	S···N	0.008	+0.028	+0.001	-0.005	+0.006
	C-S	0.216	+0.508	-0.224	-0.575	+0.351
**2a**	S···N	0.008	+0.027	+0.001	-0.005	+0.006
	C-S	0.217	+0.280	-0.238	-0.546	+0.308
**2b**	S···N	0.009	+0.029	+0.001	-0.005	+0.006
	C-S	0.217	+0.284	-0.238	-0.546	+0.309
**3a**	S···N	0.008	+0.028	+0.001	-0.005	+0.006
	C-S	0.218	+0.247	-0.241	-0.545	+0.303
**3b**	S···N	0.009	+0.030	+0.001	-0.005	+0.006
	C-S	0.218	+0.252	-0.241	-0.546	+0.304
**4a**	Se···N	0.009	+0.030	+0.001	-0.005	+0.006
	C-Se	0.187	+0.199	-0.153	-0.355	+0.203
**4b**	Se···N	0.010	+0.033	+0.001	-0.006	+0.007
	C-Se	0.186	+0.201	-0.152	-0.355	+0.203
**5a**	Se···N	0.009	+0.031	+0.001	-0.005	+0.006
	C-Se	0.184	+0.090	-0.151	-0.325	+0.174
**5b**	Se···N	0.010	+0.034	+0.001	-0.006	+0.007
	C-Se	0.184	+0.093	-0.151	-0.325	+0.174
**6a**	Se···N	0.009	+0.031	+0.001	-0.005	+0.007
	C-Se	0.184	+0.074	-0.152	-0.321	+0.170
**6b**	Se···N	0.011	+0.035	+0.001	-0.006	+0.007
	C-Se	0.184	+0.077	-0.151	-0.322	+0.170
**7a**	S···N	0.005	+0.015	+0.001	-0.002	+0.003
	Si-S	0.115	+0.348	-0.059	-0.205	+0.146
**10a**	Se···N	0.006	+0.019	+0.001	-0.003	+0.004
	Si-Se	0.106	+0.169	-0.063	-0.167	+0.105
**10b**	Se···N	0.007	+0.021	+0.001	-0.004	+0.004
	Si-Se	0.106	+0.167	-0.062	-0.167	+0.104
**11a**	Se···N	0.006	+0.018	+0.001	-0.003	+0.004
	Si-Se	0.107	+0.169	-0.063	-0.168	+0.105
**11b**	Se···N	0.006	+0.019	+0.001	-0.003	+0.004
	Si-Se	0.107	+0.170	-0.063	-0.169	+0.106
**12a**	Se···N	0.006	+0.017	+0.001	-0.003	+0.004
	Si-Se	0.107	+0.168	-0.063	-0.168	+0.105
**12b**	Se···N	0.006	+0.018	+0.001	-0.003	+0.004
	Si-Se	0.107	+0.167	-0.063	-0.168	+0.105
**13a**	S···N	0.005	+0.017	+0.001	-0.003	+0.004
	Ge-S	0.129	+0.078	-0.080	-0.180	+0.100
**15b**	S···N	0.002	+0.005	+0.000	-0.001	+0.001
	Ge-S	0.127	+0.075	-0.079	-0.176	+0.098

**16a**	Se···N	0.007	+0.021	+0.001	-0.004	+0.005
	Ge-Se	0.115	-0.011	-0.069	-0.135	+0.066
**16b**	Se···N	0.007	+0.023	+0.001	-0.004	+0.005
	Ge-Se	0.115	-0.011	-0.069	-0.135	+0.066
**17a**	Se···N	0.006	+0.020	+0.001	-0.003	+0.004
	Ge-Se	0.115	-0.011	-0.069	-0.135	+0.066
**17b**	Se···N	0.007	+0.021	+0.001	-0.004	+0.004
	Ge-Se	0.115	-0.012	-0.069	-0.135	+0.066
**18a**	Se···N	0.006	+0.019	+0.001	-0.003	+0.004
	Ge-Se	0.114	-0.012	-0.069	-0.135	+0.066
**18b**	Se···N	0.006	+0.020	+0.001	-0.003	+0.004
	Ge-Se	0.114	-0.013	-0.069	-0.135	+0.066

Table S2. AIM descriptors of the calculated complexes in acetone. Bond critical point (BCP) properties: electron density ρ, Laplacian of electron density $\nabla^2\rho$ and total electron energy H and potential electron density energy V as well as kinetic electron density energy G, were obtained at the MP2/ aug-cc-pVDZ level. Data in atomic units.

	interaction	ρ	$\nabla^2\rho$	H	V	G
**1a**	S···N	0.008	+0.025	+0.001	-0.004	+0.005
	C-S	0.216	+0.499	-0.225	-0.574	+0.349
**1b**	S···N	0.008	+0.026	+0.001	-0.005	+0.006
	C-S	0.216	+0.499	-0.225	-0.574	+0.349
**2a**	S···N	0.008	+0.026	+0.001	-0.004	+0.005
	C-S	0.217	+0.265	-0.239	-0.543	+0.305
**2b**	S···N	0.008	+0.027	+0.001	-0.005	+0.006
	C-S	0.217	+0.267	-0.239	-0.544	+0.305
**3a**	S···N	0.008	+0.026	+0.001	-0.005	+0.006
	C-S	0.218	+0.230	-0.242	-0.542	+0.300
**3b**	S···N	0.008	+0.028	+0.001	-0.005	+0.006
	C-S	0.218	+0.232	-0.242	-0.542	+0.300
**4a**	Se···N	0.009	+0.031	+0.001	-0.005	+0.007
	C-Se	0.187	+0.197	-0.153	-0.356	+0.203
**4b**	Se···N	0.010	+0.033	+0.001	-0.006	+0.007
	C-Se	0.187	+0.198	-0.153	-0.355	+0.202
**5a**	Se···N	0.009	+0.030	+0.001	-0.005	+0.006
	C-Se	0.185	+0.085	-0.152	-0.325	+0.173
**5b**	Se···N	0.010	+0.033	+0.001	-0.006	+0.007
	C-Se	0.185	+0.087	-0.152	-0.325	+0.173
**6a**	Se···N	0.009	+0.031	+0.001	-0.005	+0.007
	C-Se	0.185	+0.068	-0.152	-0.321	+0.169
	Se···N	0.010	+0.033	+0.001	-0.006	+0.007
**6b**	C-Se	0.184	+0.070	-0.152	-0.321	+0.169
**7a**	S···N	0.002	+0.006	+0.000	-0.001	+0.001
	Si-S	0.113	+0.340	-0.057	-0.200	+0.142
**7b**	S···N	0.002	+0.006	+0.000	-0.001	+0.001

	Si-S	0.113	+0.340	-0.057	-0.200	+0.142
**8a**	S···N	0.002	+0.006	+0.000	-0.001	+0.001
	Si-S	0.112	+0.335	-0.057	-0.199	+0.141
**8b**	S···N	0.002	+0.006	+0.000	-0.001	+0.001
	Si-S	0.113	+0.335	-0.056	-0.199	+0.141
**9a**	S···N	0.002	+0.005	+0.000	-0.001	+0.001
	Si-S	0.112	+0.329	-0.057	-0.196	+0.139
**9b**	S···N	0.002	+0.006	+0.000	-0.001	+0.001
	Si-S	0.112	+0.329	-0.057	-0.197	+0.139
**10a**	Se···N	0.005	+0.014	+0.001	-0.002	+0.003
	Si-Se	0.105	+0.166	-0.061	-0.164	+0.103
**10b**	Se···N	0.005	+0.014	+0.001	-0.002	+0.003
	Si-Se	0.105	+0.167	-0.061	-0.164	+0.103
**11a**	Se···N	0.004	+0.013	+0.001	-0.002	+0.003
	Si-Se	0.105	+0.164	-0.061	-0.164	+0.102
**11b**	Se···N	0.004	+0.013	+0.001	-0.002	+0.003
	Si-Se	0.105	+0.164	-0.061	-0.164	+0.102
**12a**	Se···N	0.004	+0.012	+0.001	-0.002	+0.003
	Si-Se	0.104	+0.160	-0.061	-0.162	+0.101
**12b**	Se···N	0.004	+0.012	+0.000	-0.002	+0.003
	Si-Se	0.104	+0.160	-0.061	-0.162	+0.101
**13a**	S···N	0.003	+0.008	+0.000	-0.001	+0.002
	Ge-S	0.127	+0.081	-0.078	-0.176	+0.098
**13b**	S···N	0.002	+0.007	+0.000	-0.001	+0.001
	Ge-S	0.127	+0.082	-0.078	-0.176	+0.098
**14a**	S···N	0.002	+0.007	+0.000	-0.001	+0.001
	Ge-S	0.126	+0.080	-0.077	-0.174	+0.097
**14b**	S···N	0.002	+0.006	+0.000	-0.001	+0.001
	Ge-S	0.126	+0.080	-0.077	-0.174	+0.097
**15a**	S···N	0.002	+0.006	+0.000	-0.001	+0.001
	Ge-S	0.125	+0.079	-0.076	-0.172	+0.096
**15b**	S···N	0.002	+0.006	+0.000	-0.001	+0.001
	Ge-S	0.125	+0.079	-0.076	-0.172	+0.096
**16a**	Se···N	0.005	+0.016	+0.001	-0.003	+0.003
	Ge-Se	0.113	-0.010	-0.067	-0.132	+0.065
**16b**	Se···N	0.005	+0.016	+0.001	-0.003	+0.003
	Ge-Se	0.113	-0.010	-0.067	-0.132	+0.065
**17a**	Se···N	0.005	+0.014	+0.001	-0.002	+0.003
	Ge-Se	0.113	-0.010	-0.067	-0.131	+0.064
**17b**	Se···N	0.005	+0.014	+0.001	-0.002	+0.003
	Ge-Se	0.113	-0.010	-0.067	-0.131	+0.064
**18a**	Se···N	0.004	+0.013	+0.001	-0.002	+0.003
	Ge-Se	0.112	-0.010	-0.066	-0.129	+0.063
**18b**	Se···N	0.004	+0.013	+0.001	-0.002	+0.003
	Ge-Se	0.112	-0.011	-0.066	-0.130	+0.063

Tab. S3. AIM descriptors of the calculated complexes in water. Bond critical point (BCP) properties: electron density ρ, Laplacian of electron density $\nabla^2\rho$ and total electron energy H and potential electron density energy V as well as kinetic electron density energy G, were obtained at the MP2/ aug-cc-pVDZ level. Data in atomic units.

	interaction	ρ	$\nabla^2\rho$	H	V	G
**1a**	S···N	0.008	+0.025	+0.001	-0.004	+0.005
	C-S	0.216	+0.498	-0.225	-0.574	+0.349
**1b**	S···N	0.008	+0.026	+0.001	-0.004	+0.006
	C-S	0.216	+0.498	-0.225	-0.574	+0.349
**2a**	S···N	0.008	+0.026	+0.001	-0.004	+0.005
	C-S	0.217	+0.264	-0.239	-0.543	+0.305
**2b**	S···N	0.008	+0.027	+0.001	-0.005	+0.006
	C-S	0.217	+0.265	-0.239	-0.544	+0.305
**3a**	S···N	0.008	+0.026	+0.001	-0.004	+0.005
	C-S	0.218	+0.228	-0.242	-0.541	+0.299
**3b**	S···N	0.008	+0.027	+0.001	-0.005	+0.006
	C-S	0.218	+0.230	-0.242	-0.542	+0.299
**4a**	Se···N	0.009	+0.031	+0.001	-0.005	+0.007
	C-Se	0.187	+0.197	-0.153	-0.356	+0.203
**4b**	Se···N	0.010	+0.033	+0.001	-0.006	+0.007
	C-Se	0.187	+0.197	-0.153	-0.355	+0.202
**5a**	Se···N	0.009	+0.030	+0.001	-0.005	+0.006
	C-Se	0.185	+0.085	-0.152	-0.325	+0.173
**5b**	Se···N	0.010	+0.033	+0.001	-0.006	+0.007
	C-Se	0.185	+0.086	-0.152	-0.325	+0.173
**6a**	Se···N	0.009	+0.031	+0.001	-0.005	+0.007
	C-Se	0.185	+0.067	-0.152	-0.321	+0.169
**6b**	Se···N	0.010	+0.033	+0.001	-0.006	+0.007
	C-Se	0.185	+0.069	-0.152	-0.321	+0.169
**7a**	S···N	0.002	+0.006	+0.000	-0.001	+0.001
	Si-S	0.113	+0.339	-0.057	-0.199	+0.142
**7b**	S···N	0.002	+0.006	+0.000	-0.001	+0.001
	Si-S	0.113	+0.339	-0.057	-0.199	+0.142
**8a**	S···N	0.002	+0.006	+0.000	-0.001	+0.001
	Si-S	0.112	+0.334	-0.057	-0.198	+0.141
**8b**	S···N	0.002	+0.006	+0.000	-0.001	+0.001
	Si-S	0.112	+0.334	-0.057	-0.198	+0.141
**9a**	S···N	0.002	+0.006	+0.000	-0.001	+0.001
	Si-S	0.111	+0.327	-0.057	-0.196	+0.139
**9b**	S···N	0.002	+0.005	+0.000	-0.001	+0.001
	Si-S	0.111	+0.327	-0.057	-0.196	+0.139
**10a**	Se···N	0.005	+0.014	+0.001	-0.002	+0.003
	Si-Se	0.105	+0.166	-0.061	-0.164	+0.103
**10b**	Se···N	0.005	+0.014	+0.001	-0.002	+0.003
	Si-Se	0.105	+0.166	-0.061	-0.164	+0.103

**11a**	Se···N	0.004	+0.013	+0.001	-0.002	+0.003
	Si-Se	0.105	+0.163	-0.061	-0.163	+0.102
**11b**	Se···N	0.004	+0.012	+0.001	-0.002	+0.003
	Si-Se	0.105	+0.163	-0.061	-0.163	+0.102
**12a**	Se···N	0.004	+0.012	+0.000	-0.002	+0.002
	Si-Se	0.104	+0.159	-0.061	-0.162	+0.101
**12b**	Se···N	0.004	+0.012	+0.000	-0.002	+0.002
	Si-Se	0.104	+0.159	-0.061	-0.162	+0.101
**13a**	S···N	0.002	+0.007	+0.000	-0.001	+0.002
	Ge-S	0.126	+0.082	-0.077	-0.175	+0.098
**13b**	S···N	0.002	+0.007	+0.000	-0.001	+0.001
	Ge-S	0.126	+0.082	-0.077	-0.175	+0.098
**14a**	S···N	0.002	+0.006	+0.000	-0.001	+0.001
	Ge-S	0.125	+0.080	-0.077	-0.173	+0.097
**14b**	S···N	0.002	+0.006	+0.000	-0.001	+0.001
	Ge-S	0.125	+0.080	-0.077	-0.173	+0.097
**15a**	S···N	0.002	+0.006	+0.000	-0.001	+0.001
	Ge-S	0.124	+0.079	-0.076	-0.171	+0.095
**15b**	S···N	0.002	+0.006	+0.000	-0.001	+0.001
	Ge-S	0.124	+0.079	-0.076	-0.171	+0.095
**16a**	Se···N	0.005	+0.015	+0.001	-0.003	+0.003
	Ge-Se	0.113	-0.010	-0.067	-0.132	+0.065
**16b**	Se···N	0.005	+0.015	+0.001	-0.003	+0.003
	Ge-Se	0.113	-0.010	-0.067	-0.132	+0.065
**17a**	Se···N	0.005	+0.013	+0.001	-0.002	+0.003
	Ge-Se	0.112	-0.010	-0.067	-0.131	+0.064
**17b**	Se···N	0.005	+0.013	+0.001	-0.002	+0.003
	Ge-Se	0.112	-0.010	-0.067	-0.131	+0.064
**18a**	Se···N	0.004	+0.012	+0.000	-0.002	+0.003
	Ge-Se	0.111	-0.010	-0.066	-0.129	+0.063
**18b**	Se···N	0.004	+0.012	+0.000	-0.0021	+0.003
	Ge-Se	0.111	-0.010	-0.066	-0.129	+0.063

Table S4. LMOEDA/MP2/aug-cc-pVDZ decomposition of the interaction energy in acetone of complexes into electrostatic (E_{es}), Pauli repulsion (E_{Pauli}), polarization (E_{pol}) and dispersion (E_{disp}) components. All quantities in kcal/mol.

	E_{es}	E_{Pauli}[a]	E_{pol}	%[b]	E_{disp}	%	E_{int}
acetone							
**1a**	5.55	1.93	-7.32	86	-1.22	14	-1.06
**1b**	6.05	2.15	-8.11	86	-1.31	14	-1.22
**2a**	6.13	2.05	-7.80	84	-1.45	16	-1.08
**2b**	6.72	2.31	-8.71	85	-1.58	15	-1.25
**3a**	6.23	2.13	-7.96	84	-1.50	16	-1.10
**3b**	6.82	2.42	-8.86	84	-1.64	16	-1.27
**4a**	4.35	3.03	-7.39	83	-1.54	17	-1.55
**4b**	4.47	3.57	-8.17	83	-1.68	17	-1.82

**5a**	5.05	3.10	-7.88	82	-1.78	18	-1.51
**5b**	5.30	3.68	-8.77	82	-1.98	18	-1.77
**6a**	5.18	3.19	-8.05	81	-1.83	19	-1.51
**6b**	5.40	3.80	-8.96	81	-2.04	19	-1.77

[a] $E_{Pauli}=\sum(E_{ex}+E_{rep})$, [b] percentage contribution to total attractive interactions ($E_{pol}+E_{disp}$),

Table S5. LMOEDA/MP2/aug-cc-pVDZ decomposition of the interaction energy in acetone of complexes into electrostatic (E_{es}), Pauli repulsion (E_{Pauli}), polarization (E_{pol}) and dispersion (E_{disp}) components. All quantities in kcal/mol.

	E_{es}	E_{Pauli}[a]	E_{pol}	%[b]	E_{disp}	%	Eint
			acetone				
**7a**	17.81	0.16	-17.65	97	-0.55	3	-0.22
**7b**	19.33	0.14	-19.13	97	-0.56	3	-0.23
**8a**	19.45	0.13	-19.26	97	-0.52	3	-0.20
**8b**	21.08	0.13	-20.85	97	-0.56	3	-0.20
**9a**	21.07	0.13	-20.86	98	-0.51	2	-0.18
**9b**	22.79	0.13	-22.53	98	-0.56	2	-0.18
**10a**	16.28	0.97	-16.43	93	-1.19	7	-0.37
**10b**	17.73	0.98	-17.86	93	-1.26	7	-0.41
**11a**	18.23	0.84	-18.23	94	-1.12	6	-0.28
**11b**	19.82	0.83	-19.77	94	-1.19	6	-0.31
**12a**	20.09	0.75	-19.98	95	-1.07	5	-0.21
**12b**	21.82	0.76	-21.65	95	-1.15	5	-0.22
**13a**	20.38	0.29	-20.17	97	-0.73	3	-0.24
**13b**	21.93	0.2	-21.70	97	-0.69	3	-0.25
**14a**	22.01	0.19	-21.78	97	-0.61	3	-0.20
**14b**	23.65	0.14	-23.40	97	-0.61	3	-0.21
**15a**	23.57	0.14	-23.34	98	-0.55	2	-0.19
**15b**	25.37	0.14	-25.09	98	-0.60	2	-0.18
**16a**	18.37	1.15	-18.61	93	-1.31	7	-0.39
**16b**	19.78	1.19	-20.00	93	-1.42	7	-0.44
**17a**	20.34	0.94	-20.38	94	-1.21	6	-0.31
**17b**	22.02	0.94	-22.00	94	-1.30	6	-0.34
**18a**	22.23	0.82	-22.13	95	-1.15	5	-0.23
**18b**	24.01	0.82	-23.85	95	-1.24	5	-0.25

[a] $E_{Pauli}=\sum(E_{ex}+E_{rep})$, [b] percentage contribution to total attractive interactions ($E_{pol}+E_{disp}$),

Table S6. MEP (kcal/mol of each monomer at a point along the extension of the T=Ch bond axis, at a distance from Ch that corresponds to its vdW radius in three different phases.

monomer	gas	acetone	water
7	-9.23	-18.01	-18.91
8	-10.81	-24.41	-25.36
9	-17.59	-30.30	-31.35
11	4.31	-8.58	-9.51

13	-5.26	-19.71	-20.81
14	-10.03	-25.80	-26.96
15	-14.35	-31.47	-32.75

Table S7. Atomic polar tensor (APT) charges (e) on chalcogen atom in monomers in vacuum, acetone and water solvents . Calculations performed at the MP2/aug-cc-pVDZ level of theory.

	gas	acetone	water
**1**	-0.222	-0.290	-0.295
**2**	-0.257	-0.362	-0.370
**3**	-0.291	-0.422	-0.431
**4**	-0.110	-0.140	-0.142
**5**	-0.113	-0.141	-0.142
**6**	-0.141	-0.183	-0.185
**7**	-0.442	-0.754	-0.778
**8**	-0.523	-0.859	-0.883
**9**	-0.589	-0.946	-0.972
**10**	-0.357	-0.652	-0.675
**11**	-0.443	-0.765	-0.790
**12**	-0.512	-0.856	-0.882
**13**	-0.444	-0.807	-0.835
**14**	-0.525	-0.907	-0.936
**15**	-0.595	-0.994	-1.023
**16**	-0.363	-0.708	-0.736
**17**	-0.449	-0.818	-0.847
**18**	-0.521	-0.907	-0.936

Table S8. Coordinates of monomers.

	gas	acetone	water
**1**	6 -0.447917 0.526927 0.000000<br>16 0.354649 1.918495 -0.000000<br>9 -1.773321 0.373494 -0.000000<br>9 0.083038 -0.697134 -0.000000	6 -0.448313 0.526207 -0.000000<br>16 0.354333 1.917977 0.000000<br>9 -1.773086 0.374194 0.000000<br>9 0.083515 -0.696596 0.000000	6 -0.448347 0.526143 0.000000<br>16 0.354305 1.917937 -0.000000<br>9 -1.773063 0.374255 -0.000000<br>9 0.083554 -0.696552 -0.000000
**2**	6 -0.454817 0.514962 -0.000000<br>16 0.354635 1.918473 0.000000<br>17 -2.196796 0.377247 0.000000<br>17 0.298367 -1.061797 0.000000	6 -0.455389 0.513991 0.000000<br>16 0.354222 1.917762 -0.000000<br>17 -2.196544 0.378231 -0.000000<br>17 0.299100 -1.061100 -0.000000	6 -0.455431 0.513918 0.000000<br>16 0.354188 1.917706 -0.000000<br>17 -2.196519 0.378304 -0.000000<br>17 0.299150 -1.061044 -0.000000
**3**	6 -0.453516 0.517218 0.000000<br>16 0.355721 1.920358 -0.000000<br>35 -2.356651 0.375780 -0.000000<br>35 0.377154 -1.200896 -0.000000	6 -0.454186 0.516092 -0.000000<br>16 0.355302 1.919638 0.000000<br>35 -2.356502 0.376940 0.000000<br>35 0.378094 -1.200211 0.000000	6 -0.454237 0.516005 -0.000000<br>16 0.355269 1.919581 0.000000<br>35 -2.356480 0.377023 0.000000<br>35 0.378155 -1.200151 0.000000
**4**	6 -0.448753 0.525478 -0.000000<br>34 0.422415 2.035992 0.000000<br>9 -1.774479 0.374772 0.000000<br>9 0.084724 -0.697496 0.000000	6 -0.448550 0.525778 -0.000000<br>34 0.422231 2.035724 0.000000<br>9 -1.774637 0.374864 0.000000<br>9 0.084863 -0.697620 0.000000	6 -0.448546 0.525791 -0.000000<br>34 0.422217 2.035700 0.000000<br>9 -1.774647 0.374877 0.000000<br>9 0.084881 -0.697623 0.000000
**5**	6 -0.457371 0.510534 0.000000<br>34 0.422036 2.035338 -0.000000<br>17 -2.196221 0.379932 -0.000000<br>17 0.300402 -1.059955 -0.000000	6 -0.457092 0.510999 -0.000000<br>34 0.421624 2.034678 0.000000<br>17 -2.196578 0.380297 0.000000<br>17 0.300892 -1.060126 0.000000	6 -0.457071 0.511043 -0.000000<br>34 0.421600 2.034624 0.000000<br>17 -2.196603 0.380312 0.000000<br>17 0.300920 -1.060131 0.000000

**6**	6	-0.456434	0.512158	-0.000000	6	-0.456339	0.512330	-0.000000	6	-0.456326	0.512348	-0.000000
	35	-2.354953	0.378132	0.000000	35	-2.355402	0.378780	0.000000	35	-2.355422	0.378825	0.000000
	35	0.378339	-1.198249	0.000000	35	0.379127	-1.198316	0.000000	35	0.379175	-1.198320	0.000000
	34	0.423213	2.037381	0.000000	34	0.422779	2.036629	0.000000	34	0.422738	2.036570	0.000000
**7**	1	0.228855	-0.742407	-0.000000	1	0.243338	-0.739018	-0.000000	1	0.244450	-0.738797	-0.000000
	1	-1.885750	0.476994	-0.000000	1	-1.889982	0.491353	-0.000000	1	-1.890329	0.492450	-0.000000
	14	-0.403538	0.604136	0.000000	14	-0.411198	0.590750	0.000000	14	-0.411798	0.589691	0.000000
	16	0.584000	2.316657	-0.000000	16	0.581410	2.312296	-0.000000	16	0.581244	2.312036	-0.000000
**8**	1	-1.799945	0.345470	-0.015197	1	-1.802970	0.365366	-0.000742	1	-1.803116	0.365892	-0.000051
	6	0.507812	-1.179368	-0.011145	6	0.514961	-1.181424	-0.009035	6	0.515243	-1.181410	-0.009094
	1	0.180522	-1.759401	0.869141	1	0.175534	-1.758832	0.867937	1	0.174997	-1.758495	0.867731
	1	0.193514	-1.742394	-0.907102	1	0.202324	-1.734265	-0.911628	1	0.202803	-1.733674	-0.912066
	1	1.602978	-1.088505	-0.002217	1	1.609299	-1.088237	0.007849	1	1.609609	-1.088332	0.008347
	14	-0.316748	0.507333	-0.001621	14	-0.321173	0.486599	-0.000043	14	-0.321569	0.485576	-0.000014
	16	0.576680	2.272318	0.022273	16	0.570781	2.268025	0.007637	16	0.570788	2.267675	0.007122
**9**	6	0.512631	-1.161680	-0.018186	6	0.521472	-1.163330	-0.014766	6	0.521725	-1.163326	-0.014746
	1	0.166332	-1.739354	0.856361	1	0.172592	-1.733579	0.863227	1	0.172378	-1.733164	0.863286
	1	0.184814	-1.709548	-0.918702	1	0.189727	-1.708986	-0.914772	1	0.189573	-1.708326	-0.914985
	1	1.609934	-1.109133	-0.005853	1	1.618012	-1.105099	-0.003409	1	1.618318	-1.105174	-0.003353
	6	-2.126437	0.444706	-0.020885	6	-2.131592	0.453386	-0.016759	6	-2.131711	0.453632	-0.016704
	1	-2.461184	-0.099107	-0.921308	1	-2.463163	-0.096454	-0.914247	1	-2.462597	-0.096542	-0.914180
	1	-2.481805	-0.127289	0.853758	1	-2.478527	-0.113697	0.864051	1	-2.478122	-0.113578	0.864159
	1	-2.583790	1.443557	-0.010301	1	-2.582932	1.454428	-0.010752	1	-2.583225	1.454663	-0.010869
	14	-0.247898	0.559919	0.002054	14	-0.262217	0.536004	0.000876	14	-0.262874	0.534875	0.000886
	16	0.782255	2.252362	0.041154	16	0.777858	2.242486	0.033566	16	0.777765	2.242097	0.033521
**10**	1	0.229003	-0.739329	0.000000	1	0.243049	-0.736052	0.000000	1	0.243049	-0.736052	-0.000000
	1	-1.883214	0.478489	0.000000	1	-1.887434	0.492261	0.000000	1	-1.887434	0.492261	-0.000000
	14	-0.401045	0.608542	-0.000000	14	-0.408482	0.595692	0.000000	14	-0.408482	0.595692	0.000000
	34	0.648981	2.429754	0.000000	34	0.646594	2.425555	0.000000	34	0.646594	2.425555	-0.000000
**11**	1	-1.795758	0.348267	-0.014073	1	-1.797830	0.366350	-0.000661	1	-1.798025	0.366616	0.000053
	6	0.508526	-1.179497	-0.011627	6	0.515636	-1.181288	-0.009142	6	0.515872	-1.181331	-0.009204
	1	0.180103	-1.758261	0.869132	1	0.174093	-1.757294	0.868062	1	0.173505	-1.757044	0.867834
	1	0.192382	-1.740694	-0.908186	1	0.201390	-1.732394	-0.912370	1	0.201872	-1.731869	-0.912826
	1	1.603971	-1.091848	-0.003209	1	1.610315	-1.092068	0.007975	1	1.610570	-1.092174	0.008493
	14	-0.312447	0.511017	-0.001870	14	-0.315674	0.491505	-0.000063	14	-0.316105	0.490432	-0.000031
	34	0.636951	2.388125	0.021697	34	0.630668	2.384405	0.007695	34	0.630909	2.384587	0.007177
**12**	6	0.512980	-1.158206	-0.017586	6	0.521355	-1.159670	-0.014138	6	0.521524	-1.159727	-0.014052
	1	0.165493	-1.733229	0.858419	1	0.169901	-1.728185	0.864135	1	0.169675	-1.727928	0.864213
	1	0.182833	-1.705475	-0.917784	1	0.187765	-1.703553	-0.914770	1	0.187383	-1.702817	-0.914958
	1	1.610463	-1.109286	-0.006075	1	1.618105	-1.105688	-0.002552	1	1.618321	-1.105844	-0.002587
	6	-2.125312	0.443763	-0.020341	6	-2.130060	0.451856	-0.016329	6	-2.130188	0.451997	-0.016212
	1	-2.457235	-0.102547	-0.920468	1	-2.457959	-0.099072	-0.914741	1	-2.457228	-0.099314	-0.914653
	1	-2.476563	-0.128858	0.855736	1	-2.473208	-0.117779	0.864501	1	-2.472937	-0.117753	0.864610
	1	-2.588144	1.440099	-0.010577	1	-2.587344	1.450201	-0.009738	1	-2.587629	1.450322	-0.009934
	14	-0.245813	0.565664	0.001308	14	-0.259526	0.542620	0.000482	14	-0.260179	0.541504	0.000447
	34	0.847526	2.366338	0.040258	34	0.842850	2.356926	0.031606	34	0.843136	2.357217	0.031584
**13**	1	0.255810	-0.786856	0.000000	1	0.273570	-0.782524	0.000000	1	0.275027	-0.782205	0.000000
	1	-1.937733	0.478027	0.000000	1	-1.942839	0.495629	0.000000	1	-1.943298	0.497039	0.000000
	32	-0.408752	0.595091	-0.000000	32	-0.418235	0.578615	-0.000000	32	-0.419041	0.577238	-0.000000
	16	0.614243	2.369119	0.000000	16	0.611071	2.363660	0.000000	16	0.610879	2.363307	0.000000
**14**	1	-1.875470	0.383015	-0.000183	1	-1.880187	0.408953	-0.000584	1	-1.880537	0.410256	0.000103
	6	0.523605	-1.219567	-0.011412	6	0.531398	-1.222525	-0.009314	6	0.531951	-1.222633	-0.009397
	1	0.192905	-1.801898	0.864236	1	0.199682	-1.796378	0.871046	1	0.199232	-1.796069	0.870845
	1	0.226044	-1.772347	-0.917709	1	0.226046	-1.771574	-0.914842	1	0.226555	-1.770970	-0.915325
	1	1.615593	-1.103097	0.009801	1	1.622202	-1.099710	0.007709	1	1.622724	-1.099736	0.008298
	32	-0.342727	0.525533	-0.000015	32	-0.349972	0.501466	-0.000139	32	-0.350701	0.499768	-0.000114
	16	0.604863	2.343812	0.009414	16	0.599586	2.336999	0.008101	16	0.599530	2.336615	0.007566
**15**	6	0.561293	-1.213801	-0.018401	6	0.572448	-1.216508	-0.015198	6	0.573724	-1.217270	-0.011490
	1	0.221494	-1.787786	0.859486	1	0.233755	-1.781583	0.868461	1	0.219800	-1.787123	0.862909
	1	0.239554	-1.757959	-0.921744	1	0.244711	-1.760286	-0.916095	1	0.263012	-1.756190	-0.921400
	1	1.656337	-1.133239	-0.005925	1	1.666291	-1.126815	-0.007164	1	1.667130	-1.125479	0.015699
	6	-2.195086	0.463996	-0.021224	6	-2.202244	0.474253	-0.017795	6	-2.203491	0.475058	-0.014067
	1	-2.529317	-0.072655	-0.924523	1	-2.533681	-0.068067	-0.918199	1	-2.538356	-0.049774	-0.923585
	1	-2.550174	-0.100593	0.856706	1	-2.549723	-0.084509	0.866427	1	-2.548325	-0.099617	0.860741
	1	-2.626522	1.473705	-0.010407	1	-2.624246	1.487388	-0.011894	1	-2.623504	1.488768	0.011073
	32	-0.245178	0.564389	0.002337	32	-0.262471	0.535685	0.001106	32	-0.264341	0.532537	-0.003527
	16	0.822453	2.318375	0.041787	16	0.816388	2.305599	0.037366	16	0.815580	2.304247	0.010663
**16**	1	0.255064	-0.782497	-0.000000	1	0.270689	-0.778722	0.000000	1	0.272001	-0.778447	-0.000000
	1	-1.933648	0.479406	-0.000000	1	-1.938164	0.494887	0.000000	1	-1.938582	0.496156	-0.000000
	32	-0.405134	0.601449	0.000000	32	-0.413501	0.586889	-0.000000	32	-0.414231	0.585635	0.000000
	34	0.677444	2.479098	-0.000000	34	0.674701	2.474402	-0.000000	34	0.674539	2.474112	-0.000000
**17**	1	-1.869752	0.383991	0.000730	1	-1.873568	0.407597	-0.000122	1	-1.873636	0.407828	0.000226
	6	0.524040	-1.218455	-0.012068	6	0.531929	-1.221167	-0.009448	6	0.532190	-1.221190	-0.009501

	1	0.191569	-1.799295	0.864010	1	0.196945	-1.793445	0.870876	1	0.196548	-1.793042	0.870834
	1	0.224225	-1.769446	-0.918816	1	0.224350	-1.768080	-0.915683	1	0.224448	-1.767573	-0.915987
	1	1.616490	-1.106014	0.008802	1	1.623305	-1.103688	0.008158	1	1.623591	-1.103737	0.008456
	32	-0.337451	0.530731	-0.000010	32	-0.343337	0.507985	-0.000109	32	-0.343730	0.506757	-0.000111
	34	0.664607	2.455596	0.009216	34	0.658976	2.450014	0.007827	34	0.659188	2.450173	0.007581
**18**	6	0.560351	-1.208938	-0.017816	6	0.570616	-1.211462	-0.014297	6	0.571662	-1.212034	-0.011175
	1	0.218463	-1.779891	0.861425	1	0.226663	-1.774996	0.868502	1	0.214463	-1.779538	0.863646
	1	0.235315	-1.752223	-0.920676	1	0.241924	-1.752614	-0.916672	1	0.257211	-1.748752	-0.921329
	1	1.655816	-1.133843	-0.006225	1	1.664983	-1.128138	-0.003884	1	1.665667	-1.127259	0.015550
	6	-2.192176	0.462396	-0.020694	6	-2.198760	0.471676	-0.017018	6	-2.199727	0.472365	-0.013916
	1	-2.522910	-0.077497	-0.923520	1	-2.526209	-0.071017	-0.918902	1	-2.529734	-0.055346	-0.923774
	1	-2.542407	-0.103428	0.858580	1	-2.541332	-0.091656	0.866410	1	-2.539818	-0.104896	0.861272
	1	-2.630857	1.469012	-0.010775	1	-2.628789	1.481467	-0.008874	1	-2.628364	1.482500	0.010819
	32	-0.241567	0.572658	0.001623	32	-0.257835	0.545540	0.000350	32	-0.259450	0.542800	-0.003216
	34	0.886203	2.430016	0.040969	34	0.880620	2.418860	0.032844	34	0.879970	2.417820	0.010582

Table S9. Coordinates of dimers.

	gas				acetone				water			
**1a**	6	-0.368605	0.671110	-0.000001	6	-0.375263	0.661212	0.000207	6	-0.376176	0.659540	0.000289
	9	-1.697677	0.510424	0.000005	9	-1.703650	0.519787	-0.000140	9	-1.704324	0.518966	-0.000197
	9	0.159776	-0.558956	-0.000003	9	0.142036	-0.570376	-0.000149	9	0.141037	-0.571772	-0.000209
	16	0.433001	2.063419	-0.000002	16	0.442431	2.045444	0.000137	16	0.441948	2.043642	0.000194
	7	2.043331	4.860074	-0.000000	7	2.087610	4.849182	0.000035	7	2.090190	4.850239	0.000050
	6	2.633688	5.884721	0.000000	6	2.639133	5.894348	-0.000020	6	2.640110	5.896192	-0.000028
	1	3.171643	6.818404	0.000001	1	3.142861	6.849599	-0.000070	1	3.142373	6.852389	-0.000099
**1b**	6	-0.314251	0.753737	-0.002216	6	-0.328638	0.737240	0.002352	6	-0.330750	0.732793	0.002175
	9	-1.642234	0.580080	-0.039888	9	-1.655871	0.585033	0.031267	9	-1.657465	0.579467	0.032609
	9	0.223160	-0.473108	0.031921	9	0.197632	-0.490896	-0.012918	9	0.197344	-0.494136	-0.014343
	16	0.475484	2.152707	0.001273	16	0.478316	2.127840	-0.010933	16	0.474451	2.124547	-0.011538
	7	2.046232	4.934142	0.003908	7	2.099774	4.922750	-0.015232	7	2.096607	4.927557	-0.021177
	6	2.627476	5.966956	0.001172	6	2.656297	5.969023	-0.003332	6	2.656370	5.972068	-0.006620
	6	3.348631	7.248592	-0.002315	6	3.346211	7.266379	0.011441	6	3.350276	7.267142	0.011358
	1	4.261244	7.163915	0.604120	1	4.233621	7.203531	0.655788	1	4.235118	7.200861	0.658854
	1	3.623807	7.518084	-1.031645	1	3.654558	7.530354	-1.009308	1	3.662985	7.530493	-1.008184
	1	2.708580	8.037103	0.417670	1	2.668226	8.038957	0.398874	1	2.673188	8.041417	0.396868
**2a**	6	-0.343029	0.717766	-0.000043	6	-0.348908	0.704611	0.000262	6	-0.349850	0.702692	0.000006
	17	-2.089719	0.572132	0.000006	17	-2.093918	0.587587	-0.000173	17	-2.094604	0.587831	-0.000004
	17	0.406168	-0.866826	0.000029	17	0.383350	-0.883656	-0.000185	17	0.381326	-0.885644	-0.000005
	16	0.465869	2.120767	0.000003	16	0.478843	2.098505	0.000178	16	0.479190	2.095966	0.000004
	7	2.072458	4.907319	0.000002	7	2.121575	4.896026	0.000040	7	2.125636	4.896538	0.000001
	6	2.662781	5.932132	0.000002	6	2.667748	5.944109	-0.000029	6	2.668732	5.946163	-0.000001
	1	3.200629	6.865906	0.000002	1	3.166467	6.902013	-0.000093	1	3.164727	6.905649	-0.000002
**2b**	6	-0.296222	0.789866	-0.000370	6	-0.311890	0.769848	0.000199	6	-0.314161	0.766199	0.000362
	17	-2.042894	0.629183	-0.020731	17	-2.056479	0.645022	0.034429	17	-2.058359	0.642135	0.032596
	17	0.464104	-0.790832	0.018989	17	0.426493	-0.815858	-0.028151	17	0.424628	-0.818814	-0.026199
	16	0.501789	2.198753	0.000451	16	0.510086	2.167184	-0.004721	16	0.507920	2.163689	-0.004373
	7	2.066339	4.964081	0.001527	7	2.135920	4.945978	-0.018928	7	2.137802	4.948070	-0.017788
	6	2.646908	5.997390	0.002321	6	2.684495	5.996530	-0.005497	6	2.686078	5.998781	-0.005168
	6	3.367209	7.279439	0.003308	6	3.364709	7.298973	0.010671	6	3.366026	7.301274	0.010271
	1	4.333485	7.163314	0.513868	1	4.250942	7.242924	0.657294	1	4.252750	7.245186	0.656166
	1	3.543304	7.608419	-1.030433	1	3.673773	7.564910	-1.009368	1	3.674005	7.566978	-1.010115
	1	2.772101	8.040599	0.527071	1	2.680076	8.066699	0.396075	1	2.681435	8.068709	0.396248
**3a**	6	-0.329342	0.742872	0.000012	6	-0.335821	0.729106	0.000046	6	-0.337032	0.726962	-0.000180
	35	-2.237811	0.593143	-0.000017	35	-2.241786	0.604154	-0.000030	35	-2.242577	0.602970	0.000120
	35	0.496950	-0.983973	-0.000012	35	0.478338	-0.998745	-0.000032	35	0.477205	-1.000345	0.000124
	16	0.479311	2.145071	0.000027	16	0.487096	2.125383	0.000031	16	0.486233	2.123259	-0.000123
	7	2.081692	4.923389	0.000005	7	2.128597	4.913551	0.000007	7	2.130813	4.915422	-0.000026
	6	2.672852	5.947690	-0.000004	6	2.678373	5.959771	-0.000005	6	2.679681	5.962060	0.000021
	1	3.211506	6.881005	-0.000011	1	3.180359	6.915976	-0.000016	1	3.180835	6.918866	0.000065
**3b**	6	-0.283999	0.811644	-0.000259	6	-0.300251	0.788401	-0.000017	6	-0.302631	0.785147	-0.000424
	35	-2.192784	0.645995	-0.004567	35	-2.203478	0.653857	0.100910	35	-2.205486	0.650811	0.097127

	35	0.554063	-0.911267	0.003421	35	0.518582	-0.935687	-0.086595	35	0.517467	-0.937953	-0.082779
	16	0.514078	2.219454	0.000319	16	0.516510	2.188290	-0.010627	16	0.513808	2.185492	-0.011586
	7	2.073504	4.974132	0.000916	7	2.130803	4.962615	-0.026466	7	2.134388	4.963538	-0.024850
	6	2.653437	6.007788	0.001116	6	2.685567	6.009831	-0.006255	6	2.687939	6.011404	-0.005417
	6	3.373223	7.290095	0.001940	6	3.373440	7.308063	0.018605	6	3.374367	7.310310	0.018501
	1	4.181667	7.262913	0.745983	1	4.221868	7.261364	0.714794	1	4.224480	7.263928	0.712610
	1	3.804460	7.475570	-0.991849	1	3.740909	7.548846	-0.988227	1	3.738936	7.551852	-0.989165
	1	2.680475	8.105885	0.251980	1	2.674174	8.088630	0.347878	1	2.674856	8.089679	0.349983
	6	-0.403500	0.612540	-0.000000	6	-0.403509	0.611611	0.000000	6	-0.403539	0.610748	-0.000000
	9	-1.732984	0.455364	0.000000	9	-1.732428	0.463890	0.000000	9	-1.732210	0.462765	0.000000
	9	0.127041	-0.616585	-0.000000	9	0.122563	-0.617615	-0.000000	9	0.123286	-0.617939	0.000000
**4a**	34	0.468439	2.125656	0.000000	34	0.475882	2.120180	-0.000000	34	0.475179	2.119683	-0.000000
	7	2.065537	4.896712	-0.000000	7	2.080772	4.886728	0.000000	7	2.078235	4.889000	-0.000000
	6	2.656146	5.920953	-0.000000	6	2.654132	5.919911	0.000000	6	2.654107	5.920757	-0.000000
	1	3.194478	6.854555	-0.000000	1	3.177745	6.864491	-0.000000	1	3.180100	6.864182	0.000000
	6	-0.350692	0.690021	0.000040	6	-0.346376	0.683254	0.007332	6	-0.347192	0.681967	0.006917
	9	-1.679646	0.519525	-0.000780	9	-1.671782	0.504060	-0.008466	9	-1.672521	0.503980	-0.002843
	9	0.189772	-0.535988	0.000316	9	0.207379	-0.534325	0.027073	9	0.206222	-0.535190	0.028983
	34	0.508217	2.211189	0.000635	34	0.498513	2.212025	0.002188	34	0.498312	2.210454	-0.006507
**4b**	7	2.058028	4.956517	0.000067	7	2.032490	4.976820	-0.018728	7	2.034834	4.976643	-0.020844
	6	2.639568	5.988916	-0.000014	6	2.637474	5.995505	0.003571	6	2.638455	5.996109	0.003004
	6	3.361212	7.269942	-0.000049	6	3.387535	7.258486	0.031972	6	3.387672	7.259468	0.032709
	1	3.994461	7.340204	0.895426	1	4.409440	7.071125	0.388639	1	4.409040	7.072306	0.390965
	1	3.994331	7.340209	-0.895621	1	3.426740	7.685522	-0.979374	1	3.428048	7.686524	-0.978519
	1	2.642873	8.101675	-0.000020	1	2.889213	7.966739	0.707792	1	2.887756	7.966948	0.708135
	6	-0.381281	0.651272	-0.000000	6	-0.382837	0.648353	0.000000	6	-0.381907	0.646920	-0.000000
	17	-2.124817	0.513521	0.000000	17	-2.125604	0.523422	-0.000000	17	-2.125155	0.532559	0.000000
	17	0.372171	-0.927091	0.000000	17	0.364781	-0.930863	-0.000000	17	0.356759	-0.936259	0.000000
**5a**	34	0.499187	2.177361	-0.000000	34	0.505263	2.169288	0.000000	34	0.514988	2.162623	-0.000000
	7	2.097067	4.950416	-0.000000	7	2.117947	4.940642	0.000000	7	2.135647	4.931998	0.000000
	6	2.687228	5.975077	0.000000	6	2.687611	5.976008	-0.000000	6	2.686006	5.977702	0.000000
	1	3.225602	6.908640	0.000000	1	3.207996	6.922345	-0.000000	1	3.188819	6.933654	0.000000
	6	-0.338021	0.717013	-0.000485	6	-0.343852	0.710173	0.000808	6	-0.344882	0.707073	0.000225
	17	-2.081867	0.564912	-0.024819	17	-2.086174	0.577196	0.045167	17	-2.086664	0.572275	0.044833
	17	0.426080	-0.857887	0.023080	17	0.409847	-0.866530	-0.036644	17	0.411416	-0.867954	-0.035547
	34	0.531519	2.249764	0.000191	34	0.537910	2.235310	-0.005557	34	0.534689	2.233508	-0.007716
**5b**	7	2.083532	4.989467	0.000903	7	2.114736	4.980562	-0.023176	7	2.108999	4.985925	-0.020821
	6	2.663743	6.022759	0.002072	6	2.679756	6.022185	-0.007001	6	2.677318	6.025777	-0.005621
	6	3.383660	7.304717	0.003546	6	3.380065	7.313559	0.012916	6	3.382130	7.314689	0.012739
	1	4.392741	7.164344	0.415693	1	4.259667	7.244599	0.667300	1	4.262063	7.242959	0.666358
	1	3.463784	7.687580	-1.023500	1	3.702050	7.572647	-1.004893	1	3.703907	7.571892	-1.005587
	1	2.840952	8.035542	0.619322	1	2.704119	8.092507	0.391082	1	2.709148	8.096063	0.391139
	6	-0.368235	0.673880	0.000000	6	-0.369957	0.669819	0.000000	6	-0.370465	0.668875	0.000000
	35	-2.271885	0.531059	0.000000	35	-2.272522	0.544353	-0.000000	35	-2.272735	0.543728	-0.000000
	35	0.462704	-1.044797	0.000000	35	0.452412	-1.050385	0.000000	35	0.452273	-1.050797	-0.000000
**6a**	34	0.511582	2.200629	-0.000000	34	0.519955	2.190565	-0.000000	34	0.519266	2.189708	0.000000
	7	2.106898	4.968812	-0.000000	7	2.130246	4.958289	-0.000000	7	2.127915	4.960780	0.000000
	6	2.697793	5.993049	0.000000	6	2.698219	5.994601	0.000000	6	2.698788	5.995449	-0.000000
	1	3.236300	6.926564	0.000000	1	3.216805	6.941953	0.000000	1	3.220115	6.941453	-0.000000
	6	-0.327495	0.738685	0.000017	6	-0.332964	0.726844	-0.000447	6	-0.332262	0.724629	0.000045
	35	-2.233782	0.609924	-0.000005	35	-2.232481	0.586185	0.100864	35	-2.233569	0.577887	0.014133
	35	0.487942	-0.989099	-0.000004	35	0.498224	-0.987176	-0.096903	35	0.509251	-0.986726	-0.008047
	34	0.565161	2.258320	-0.000072	34	0.546554	2.254138	-0.004529	34	0.540882	2.255523	-0.005115
**6b**	7	2.138969	4.972714	0.000047	7	2.113083	4.996197	-0.013279	7	2.099407	5.006500	-0.017770
	6	2.691917	6.020859	0.000082	6	2.682562	6.035456	-0.000139	6	2.673016	6.043435	-0.000660
	6	3.378466	7.320951	-0.000001	6	3.388306	7.323870	0.016489	6	3.384121	7.328711	0.020659
	1	4.009568	7.408478	0.895514	1	4.234785	7.270317	0.714584	1	4.319270	7.221762	0.586956
	1	4.009525	7.408389	-0.895554	1	3.760811	7.552028	-0.991429	1	3.613699	7.638511	-1.007941
	1	2.637853	8.132988	-0.000024	1	2.699246	8.116350	0.338790	1	2.752811	8.089977	0.498741
	14	-0.491023	0.452666	-0.000000	14	-0.625302	0.258649	-0.000000	14	-0.625075	0.256959	0.000000
	1	0.137440	-0.897451	0.000000	1	0.115630	-1.025466	-0.000000	1	0.112077	-1.028814	0.000000
	1	-1.973972	0.316086	0.000000	1	-2.094422	0.059422	-0.000000	1	-2.094942	0.067258	-0.000000
**7a**	16	0.494530	2.167191	-0.000000	16	0.252035	2.041684	0.000000	16	0.260683	2.036952	-0.000000
	7	2.259333	5.235611	0.000000	7	2.499079	5.435034	0.000000	7	2.498085	5.435401	-0.000000
	6	2.849494	6.261325	0.000000	6	3.019962	6.495807	-0.000000	6	3.018263	6.496396	0.000000
	1	3.386809	7.195377	0.000000	1	3.495630	7.465673	-0.000000	1	3.493522	7.466651	0.000000
			-		14	-0.866703	-0.225489	-0.001714	14	-0.867856	-0.226150	-0.001485
					1	-0.333140	-1.608752	-0.003498	1	-0.327215	-1.606115	-0.002917
					1	-2.348922	-0.195241	-0.002874	1	-2.349616	-0.198082	-0.002081
**7b**					16	0.273194	1.402076	0.001840	16	0.267361	1.405944	0.000830
					7	2.180205	5.062013	0.004613	7	2.179994	5.062161	0.002756
					6	2.770224	6.089713	0.002495	6	2.770501	6.089519	0.001591
					6	3.502080	7.364226	-0.000179	6	3.502593	7.363736	0.000139

					1	4.104528	7.444346	0.914863	1	4.104386	7.442852	0.915654
					1	4.162978	7.406964	-0.876565	1	4.163862	7.406588	-0.875916
					1	2.788254	8.198157	-0.040900	1	2.788688	8.197560	-0.040489
**8a**	-				1	-2.122357	0.063025	-0.000177	1	-2.124148	0.063479	-0.000421
					14	-0.635579	0.093842	-0.006961	14	-0.637849	0.092188	-0.006619
					6	0.094157	-1.623893	-0.005879	6	0.095488	-1.623086	-0.006014
					1	-0.263914	-2.165984	0.886172	1	-0.262582	-2.165684	0.885715
					1	-0.269313	-2.169157	-0.893826	1	-0.267135	-2.168022	-0.894493
					1	1.192336	-1.599752	-0.009353	1	1.193588	-1.597099	-0.008959
					16	0.365487	1.815941	-0.016228	16	0.362065	1.816382	-0.014696
					7	2.245295	5.485376	-0.020734	7	2.245373	5.484217	-0.020718
					6	2.770807	6.543819	-0.014106	6	2.771243	6.542342	-0.014368
					1	3.250992	7.511411	-0.008147	1	3.251867	7.509912	-0.008666
**8b**	-				1	-1.988305	0.382061	0.014244	1	-1.986505	0.390471	0.008639
					14	-0.502073	0.433080	0.002560	14	-0.500490	0.432301	0.002062
					6	0.249959	-1.275039	-0.002922	6	0.247509	-1.276666	-0.003116
					1	-0.096902	-1.823022	0.889991	1	-0.104887	-1.824782	0.887508
					1	-0.111183	-1.823525	-0.889845	1	-0.111568	-1.822240	-0.892641
					1	1.347702	-1.237256	-0.011753	1	1.345349	-1.241421	-0.007241
					16	0.475151	2.168706	-0.005807	16	0.483867	2.165363	-0.000492
					7	2.360711	5.855694	-0.008206	7	2.373184	5.848052	0.001676
					6	3.024672	6.837180	0.004011	6	3.030562	6.833947	0.008543
					6	3.849067	8.053860	0.019190	6	3.846478	8.056214	0.017235
					1	4.367867	8.158640	-0.943376	1	4.359083	8.162714	-0.948411
					1	3.208111	8.930393	0.185121	1	3.200070	8.928280	0.185299
					1	4.590111	7.985862	0.827244	1	4.592236	7.994398	0.821391
**9a**	-				6	0.393950	-1.387825	0.000815	6	0.383738	-1.399314	-0.013732
					1	0.052931	-1.946024	0.889602	1	0.042589	-1.957659	0.874901
					1	0.054206	-1.945589	-0.888709	1	0.031091	-1.948327	-0.903690
					1	1.490680	-1.330310	0.001734	1	1.480953	-1.353364	-0.020485
					14	-0.388438	0.312611	0.000856	14	-0.382215	0.307484	0.000563
					6	-2.258168	0.226482	-0.004235	6	-2.251878	0.244792	0.009304
					1	-2.594567	-0.330247	-0.895666	1	-2.600506	-0.304080	-0.882238
					1	-2.599317	-0.334225	0.882868	1	-2.592034	-0.316690	0.896249
					1	-2.711050	1.226953	-0.003243	1	-2.693196	1.250367	0.018475
					16	0.650035	2.019719	0.005954	16	0.675697	2.004397	0.006019
					7	2.565550	5.696611	0.009404	7	2.584961	5.680628	0.010361
					6	3.116285	6.742177	0.010974	6	3.114310	6.737047	0.012516
					1	3.619387	7.698049	0.012402	1	3.597975	7.703098	0.014510
**9b**	-				6	0.136699	-1.523833	0.748073	6	0.309081	-1.530469	-0.031847
					1	-0.609034	-1.946902	1.442765	1	-0.023943	-2.075724	0.867962
					1	0.259373	-2.242255	-0.080549	1	-0.078944	-2.074623	-0.909943
					1	1.094996	-1.397280	1.269380	1	1.406634	-1.512143	-0.065309
					14	-0.501557	0.099460	0.068964	14	-0.412504	0.195449	-0.007022
					6	-2.139050	-0.101840	-0.815265	6	-2.282761	0.182730	0.035700
					1	-2.020244	-0.821228	-1.643665	1	-2.661934	-0.347151	-0.854921
					1	-2.880999	-0.524485	-0.116280	1	-2.622513	-0.378600	0.922850
					1	-2.508142	0.854588	-1.209121	1	-2.696253	1.199760	0.062182
					16	0.453980	1.843398	0.263652	16	0.689198	1.864092	-0.023893
					7	2.581621	5.397018	0.491487	7	2.610598	5.551705	-0.022247
					6	3.152116	6.413684	0.704196	6	3.120447	6.621221	-0.003667
					6	3.859002	7.674666	0.969181	6	3.752280	7.947804	0.019662
					1	4.128418	7.728697	2.032739	1	3.475706	8.470177	0.945551
					1	4.771450	7.720161	0.359252	1	4.844121	7.835597	-0.023806
					1	3.207742	8.521822	0.714587	1	3.410884	8.532663	-0.845222
**10a**	14	-0.507511	0.423854	-0.000000	14	-0.552259	0.346448	0.000000	14	-0.557231	0.338183	-0.000000
	1	0.117372	-0.928583	-0.000000	1	0.069788	-1.000636	0.000000	1	0.067352	-1.007228	-0.000000
	1	-1.990613	0.282534	-0.000000	1	-2.033677	0.263798	0.000000	1	-2.038324	0.257966	0.000000
	34	0.540876	2.248677	0.000000	34	0.535685	2.157619	-0.000000	34	0.530779	2.150249	0.000000
	7	2.261718	5.239713	0.000000	7	2.358893	5.298713	-0.000000	7	2.365828	5.307416	0.000000
	6	2.851750	6.265239	0.000000	6	2.896575	6.351395	-0.000000	6	2.902185	6.360682	0.000000
	1	3.389020	7.199370	-0.000000	1	3.387607	7.313469	0.000000	1	3.392023	7.323537	-0.000000
**10b**	14	-0.489784	0.454085	0.000505	14	-0.561334	0.333997	-0.000049	14	-0.570064	0.321431	-0.000132
	1	0.133114	-0.899737	0.003437	1	0.069269	-1.009254	-0.004550	1	0.060789	-1.021128	-0.003660
	1	-1.973110	0.310642	0.001460	1	-2.042311	0.241450	0.002260	1	-2.050802	0.233590	0.000700
	34	0.559008	2.279167	-0.003863	34	0.514901	2.152182	0.002613	34	0.509341	2.138704	0.003154
	7	2.261368	5.241078	-0.001948	7	2.333635	5.298710	0.003328	7	2.347949	5.301642	0.004082
	6	2.852628	6.268809	-0.001035	6	2.908811	6.335180	0.001483	6	2.918143	6.340812	0.001846
	6	3.586083	7.543700	0.000139	6	3.621769	7.620321	-0.000828	6	3.624958	7.629237	-0.000943
	1	4.190706	7.625435	0.914313	1	4.225689	7.707857	0.912598	1	4.229216	7.719138	0.911969
	1	4.249091	7.593525	-0.875030	1	4.279569	7.674670	-0.878933	1	4.281458	7.686489	-0.879789
	1	2.876689	8.382325	-0.037978	1	2.895795	8.443915	-0.037924	1	2.894808	8.449114	-0.037229
**11a**	1	-1.876321	0.599819	-0.002242	1	-1.935178	0.510837	0.004137	1	-1.939175	0.506645	0.004287
	14	-0.383636	0.666032	0.000331	14	-0.447586	0.554412	0.006614	14	-0.452028	0.547920	0.006395

	6	0.305914	-1.085404	-0.000202	6	0.280798	-1.166944	0.007800	6	0.280108	-1.170892	0.007774
	1	-0.059584	-1.630843	0.887294	1	-0.079080	-1.710012	-0.883098	1	-0.079216	-1.714365	-0.883076
	1	-0.056596	-1.629126	-0.889974	1	1.379071	-1.144964	0.009488	1	1.378296	-1.146640	0.009441
	1	1.404970	-1.081944	0.001650	1	-0.081825	-1.709931	0.897630	1	-0.081838	-1.713952	0.897827
	34	0.700772	2.470488	0.003898	34	0.609452	2.389403	0.007803	34	0.603635	2.385034	0.007558
	7	2.443953	5.491019	0.009747	7	2.449998	5.580371	0.008036	7	2.454075	5.590657	0.008028
	6	2.939015	6.565758	0.011618	6	2.970002	6.641916	0.008250	6	2.975293	6.651500	0.008302
	1	3.389380	7.544681	0.013321	1	3.444758	7.612089	0.008447	1	3.451259	7.621269	0.008573
**11b**		-			1	-1.926822	0.544468	0.007171	1	-1.932966	0.537211	0.005893
					14	-0.438674	0.566858	-0.004008	14	-0.445347	0.559317	-0.004063
					6	0.264482	-1.165101	-0.008959	6	0.264559	-1.168920	-0.009315
					1	-0.097195	-1.702316	0.884752	1	-0.096290	-1.707483	0.883915
					1	-0.111934	-1.703319	-0.895944	1	-0.109553	-1.707728	-0.896900
					1	1.362930	-1.159156	-0.018039	1	1.362936	-1.158669	-0.017456
					34	0.644867	2.386279	-0.012474	34	0.634578	2.382314	-0.010428
					7	2.536202	5.559714	-0.012464	7	2.536346	5.566849	-0.008813
					6	3.072553	6.616644	0.004973	6	3.074523	6.622840	0.006655
					6	3.736414	7.927657	0.027237	6	3.740522	7.932668	0.026561
					1	3.656816	8.363386	1.032521	1	3.662600	8.369466	1.031478
					1	4.796412	7.808052	-0.235107	1	4.799947	7.810657	-0.236846
					1	3.254646	8.596492	-0.698912	1	3.258843	8.601138	-0.699935
**12a**	6	0.447410	-1.252372	-0.016874	6	0.430678	-1.301051	-0.000297	6	0.424659	-1.317132	-0.000273
	1	0.122893	-1.827714	0.867811	1	0.099393	-1.865687	0.888299	1	0.089151	-1.878662	0.888710
	1	0.128583	-1.819994	-0.908607	1	0.101470	-1.863924	-0.890775	1	0.091040	-1.877567	-0.890647
	1	1.543192	-1.171658	-0.013015	1	1.526266	-1.224306	0.001117	1	1.520700	-1.247520	0.000984
	14	-0.360668	0.452033	-0.012474	14	-0.385572	0.385841	0.000513	14	-0.381319	0.373654	0.000027
	6	-2.236687	0.255834	-0.019997	6	-2.254324	0.245528	-0.003317	6	-2.250361	0.251734	-0.003265
	1	-2.554123	-0.312555	-0.911737	1	-2.574561	-0.321633	-0.894386	1	-2.575168	-0.313149	-0.894054
	1	-2.560900	-0.319693	0.864680	1	-2.578020	-0.323993	0.884992	1	-2.578127	-0.314922	0.885311
	1	-2.737622	1.233754	-0.017981	1	-2.737517	1.231799	-0.002943	1	-2.724464	1.242358	-0.003064
	34	0.671922	2.289743	-0.001487	34	0.675348	2.226235	0.005277	34	0.693246	2.207816	0.003876
	7	2.398932	5.358011	0.016861	7	2.550751	5.442997	0.009398	7	2.558258	5.459404	0.009416
	6	2.983509	6.386932	0.021013	6	3.043750	6.517373	0.011431	6	3.041373	6.538144	0.011787
	1	3.515787	7.323720	0.024795	1	3.493822	7.499200	0.013446	1	3.482497	7.524220	0.013947
**12b**	6	0.434212	-1.276726	-0.000818	6	0.387575	-1.372067	-0.034639	6	0.382829	-1.383681	-0.033664
	1	0.111997	-1.848740	0.886889	1	0.059473	-1.948677	0.847417	1	0.053368	-1.958390	0.849095
	1	0.113412	-1.847891	-0.889587	1	0.042639	-1.915560	-0.931281	1	0.035106	-1.926176	-0.929808
	1	1.530054	-1.196079	0.000075	1	1.483914	-1.307528	-0.044263	1	1.479295	-1.321894	-0.044182
	14	-0.373691	0.428556	-0.000687	14	-0.409548	0.323642	0.003892	14	-0.410812	0.312587	0.004406
	6	-2.249984	0.227839	-0.001987	6	-2.279533	0.203295	0.032621	6	-2.280486	0.203072	0.031642
	1	-2.569287	-0.344269	-0.890684	1	-2.621916	-0.347470	-0.860458	1	-2.623933	-0.344685	-0.862843
	1	-2.570439	-0.344980	0.885835	1	-2.593318	-0.375722	0.918314	1	-2.597295	-0.376266	0.915978
	1	-2.752971	1.204774	-0.001931	1	-2.751942	1.194490	0.055810	1	-2.747806	1.196622	0.056106
	34	0.657038	2.267474	0.000687	34	0.670855	2.152730	0.011885	34	0.676663	2.139192	0.012910
	7	2.366992	5.320253	0.005528	7	2.560638	5.364986	0.010152	7	2.569161	5.374127	0.010141
	6	2.945359	6.355498	0.009588	6	3.065100	6.437668	0.010737	6	3.070868	6.448049	0.010588
	6	3.662746	7.639629	0.014758	6	3.689317	7.768238	0.011348	6	3.691782	7.780004	0.011276
	1	3.609993	8.092872	1.014678	1	3.402252	8.309009	0.923463	1	3.406861	8.317998	0.925665
	1	4.716949	7.479645	-0.251861	1	4.782229	7.662204	-0.020538	1	4.784748	7.676328	-0.025165
	1	3.207719	8.324634	-0.714454	1	3.352361	8.333253	-0.868429	1	3.349747	8.345604	-0.866114
**13a**	32	-0.492240	0.450344	-0.000000	32	-0.587839	0.277554	-0.000000	32	-0.613564	0.262549	0.000000
	1	0.166708	-0.936263	0.000000	1	0.059767	-1.105589	0.000000	1	0.123284	-1.074374	0.000000
	1	-2.022161	0.322930	0.000000	1	-2.114527	0.239926	-0.000000	1	-2.134553	0.131994	-0.000000
	16	0.529503	2.226451	0.000000	16	0.495810	2.030661	-0.000000	16	0.359859	2.080548	-0.000000
	7	2.254578	5.227206	0.000000	7	2.428451	5.399961	-0.000000	7	2.473178	5.411334	-0.000000
	6	2.844516	6.253030	0.000000	6	2.951578	6.459766	0.000000	6	2.990792	6.473673	0.000000
	1	3.381707	7.187107	-0.000000	1	3.429371	7.428524	0.000000	1	3.463616	7.445079	0.000000
**13b**		-			32	-0.611180	0.182222	0.000421	32	-0.612695	0.180724	0.000502
					1	0.175832	-1.126491	0.005340	1	0.185761	-1.120324	0.005559
					1	-2.125977	-0.011694	-0.002288	1	-2.126027	-0.019761	-0.001784
					16	0.287783	2.036611	-0.002320	16	0.274633	2.042189	-0.002875
					7	2.077849	5.604965	-0.001804	7	2.075911	5.607179	-0.002734
					6	2.816528	6.531703	-0.000966	6	2.816237	6.532537	-0.001363
					6	3.733958	7.679918	0.000187	6	3.735511	7.679108	0.000353
					1	4.354645	7.653918	0.906137	1	4.355815	7.651434	0.906478
					1	4.381142	7.634508	-0.886250	1	4.382582	7.632623	-0.886065
					1	3.155211	8.613365	-0.018456	1	3.158064	8.613317	-0.018071
**14a**		-			1	-2.085713	0.361462	-0.015951	1	-2.092532	0.350561	-0.015586
					32	-0.552454	0.339807	-0.010509	32	-0.560126	0.320133	-0.009616
					6	0.194560	-1.446797	-0.004112	6	0.185305	-1.466091	-0.004388
					1	-0.168991	-1.979884	0.889290	1	-0.181403	-1.999498	0.887501
					1	-0.163728	-1.984365	-0.896946	1	-0.172315	-2.001037	-0.899048
					1	1.291703	-1.408522	-0.000934	1	1.282439	-1.428941	0.001156
					16	0.531871	2.099143	-0.010155	16	0.530331	2.077508	-0.007723
					7	2.361071	5.685390	-0.004322	7	2.404915	5.715262	-0.002854

					6	2.911945	6.730895	-0.015443	6	2.929661	6.773914	-0.016058
					1	3.415175	7.686654	-0.025455	1	3.409164	7.741973	-0.027921
**14b**	-				1	-2.064421	0.409433	0.153424	1	-2.049124	0.459491	0.025828
					32	-0.535535	0.431419	0.037059	32	-0.516949	0.423003	0.005863
					6	0.259530	-1.333142	-0.027862	6	0.221410	-1.366109	-0.002291
					1	-0.024789	-1.885237	0.882528	1	-0.131801	-1.898465	0.895648
					1	-0.146057	-1.872161	-0.899383	1	-0.153551	-1.899314	-0.890852
					1	1.352597	-1.263551	-0.102291	1	1.318611	-1.333137	-0.015706
					16	0.496036	2.220464	-0.036734	16	0.580421	2.175836	-0.009781
					7	2.352996	5.885218	-0.033585	7	2.498317	5.824518	-0.017871
					6	3.024544	6.861307	-0.008378	6	3.096624	6.847239	-0.001568
					6	3.858465	8.071155	0.023023	6	3.838589	8.115533	0.018905
					1	4.370719	8.189933	-0.941428	1	4.186136	8.354458	-0.995343
					1	3.225685	8.949355	0.210206	1	3.182091	8.919447	0.378544
					1	4.605117	7.982437	0.823874	1	4.704114	8.024129	0.689077
**15a**	-				6	0.469714	-1.388153	-0.000715	6	0.469860	-1.390451	-0.000524
					1	0.160532	-1.958467	0.890349	1	0.160398	-1.958999	0.891526
					1	0.163371	-1.955927	-0.894375	1	0.162323	-1.958479	-0.893570
					1	1.558543	-1.249668	0.001227	1	1.558719	-1.252149	0.000695
					32	-0.440319	0.326658	0.000311	32	-0.440464	0.323105	-0.000970
					6	-2.375926	0.182047	-0.003212	6	-2.375586	0.186018	-0.003041
					1	-2.691108	-0.380768	-0.896864	1	-2.692305	-0.376813	-0.896084
					1	-2.694114	-0.382941	0.888003	1	-2.694112	-0.377292	0.889059
					1	-2.839761	1.176821	-0.002763	1	-2.836182	1.182300	-0.003236
					16	0.561918	2.141382	0.004932	16	0.565579	2.137873	0.000821
					7	2.484131	5.794150	0.011177	7	2.499655	5.780077	0.010808
					6	3.193123	6.739611	0.011941	6	3.190676	6.738600	0.012754
					1	3.841379	7.603635	0.012744	1	3.822923	7.614589	0.014516
**15b**	6	0.414408	-1.448196	0.048268	6	0.199111	-1.562002	0.762019	6	0.331135	-1.634760	-0.014405
	1	0.073439	-2.010062	0.933325	1	-0.523765	-2.015978	1.459075	1	-0.020291	-2.164084	0.886134
	1	0.154990	-2.036115	-0.847567	1	0.352047	-2.260856	-0.076451	1	-0.046661	-2.172919	-0.898962
	1	1.501858	-1.304669	0.095839	1	1.151600	-1.377852	1.275493	1	1.428294	-1.607747	-0.031042
	32	-0.495492	0.273855	-0.019604	32	-0.541351	0.094947	0.072810	32	-0.399745	0.162573	-0.013341
	6	-2.432088	0.071677	-0.101206	6	-2.236606	-0.132265	-0.845412	6	-2.338474	0.228688	0.022192
	1	-2.702693	-0.510138	-0.997670	1	-2.096541	-0.830118	-1.686931	1	-2.729913	-0.303222	-0.860100
	1	-2.781591	-0.485703	0.783367	1	-2.965018	-0.577287	-0.148259	1	-2.697461	-0.292302	0.924520
	1	-2.918650	1.055243	-0.136501	1	-2.610616	0.831280	-1.214880	1	-2.691472	1.267987	0.022217
	16	0.476349	2.090791	-0.012318	16	0.392820	1.932275	0.292859	16	0.784791	1.865755	-0.049506
	7	2.586660	5.593596	-0.277901	7	2.556506	5.449641	0.501140	7	2.674493	5.552547	-0.050618
	6	3.144036	6.618229	-0.066075	6	3.155922	6.449956	0.711868	6	3.149513	6.637595	-0.015747
	6	3.835277	7.889196	0.197893	6	3.898667	7.690624	0.974669	6	3.738523	7.983244	0.027962
	1	3.565343	8.257735	1.197221	1	4.076321	7.791219	2.054008	1	3.534343	8.442026	1.004905
	1	4.922420	7.737034	0.149945	1	4.862142	7.661225	0.447869	1	4.824369	7.914171	-0.122987
	1	3.541055	8.634466	-0.554020	1	3.315133	8.550864	0.619520	1	3.298653	8.602934	-0.765193
**16a**	32	-0.510375	0.418586	-0.000000	32	-0.556801	0.337883	0.000000	32	-0.560783	0.329848	0.000000
	1	0.141598	-0.972361	0.000000	1	0.104149	-1.040262	-0.000000	1	0.099115	-1.048138	0.000000
	1	-2.040486	0.282412	0.000000	1	-2.083179	0.259178	-0.000000	1	-2.086964	0.259533	-0.000000
	34	0.571373	2.299714	-0.000000	34	0.556264	2.213209	0.000000	34	0.558004	2.203023	-0.000000
	7	2.261136	5.239097	-0.000000	7	2.343547	5.305585	0.000000	7	2.349321	5.313041	-0.000000
	6	2.851197	6.264573	0.000000	6	2.896813	6.350208	-0.000000	6	2.900349	6.358758	-0.000000
	1	3.388169	7.198784	0.000000	1	3.401819	7.305002	-0.000000	1	3.403570	7.314738	0.000000
**16b**	32	-0.493247	0.449093	-0.000299	32	-0.563216	0.329186	-0.000817	32	-0.570948	0.313334	-0.001218
	1	0.157620	-0.942386	0.004887	1	0.098629	-1.041887	0.136172	1	0.097255	-1.060695	0.016217
	1	-2.023201	0.310894	-0.003337	1	-2.082878	0.238571	-0.139166	1	-2.096694	0.233084	-0.015455
	4	0.588388	2.331288	-0.002677	34	0.539918	2.210433	0.000569	34	0.536360	2.193377	-0.004603
	7	2.260985	5.240544	-0.002746	7	2.320287	5.302021	-0.000329	7	2.319034	5.315107	0.004472
	6	2.852435	6.268132	-0.001086	6	2.901767	6.335024	0.000113	6	2.903704	6.346252	0.002416
	6	3.586069	7.542843	0.000930	6	3.622438	7.615844	0.000732	6	3.628242	7.624762	-0.000011
	1	4.190162	7.624239	0.915522	1	4.291521	7.661248	0.870786	1	4.224525	7.711162	0.918501
	1	4.249726	7.592750	-0.873771	1	4.215357	7.705061	-0.919712	1	4.294386	7.668243	-0.872363
	1	2.876859	8.381631	-0.037423	1	2.901970	8.443526	0.051653	1	2.909929	8.454402	-0.047956
**17a**	1	-1.962209	0.609955	-0.002377	1	-2.012009	0.541229	0.003546	1	-2.012824	0.542214	0.003522
	32	-0.422315	0.681908	0.000282	32	-0.477581	0.564077	0.006014	32	-0.478918	0.556588	0.006024
	6	0.327279	-1.121556	-0.000201	6	0.293422	-1.215139	0.008096	6	0.290345	-1.222333	0.008033
	1	-0.024850	-1.668355	0.890171	1	-0.059356	-1.755472	-0.885478	1	-0.063715	-1.761583	-0.885664
	1	-0.021853	-1.666633	-0.892806	1	1.390128	-1.164241	0.009717	1	1.387062	-1.172653	0.009750
	1	1.424959	-1.078979	0.001686	1	-0.062036	-1.754491	0.901201	1	-0.066479	-1.760510	0.901295
	34	0.689990	2.547108	0.003954	34	0.630691	2.448860	0.006957	34	0.634185	2.440249	0.007157
	7	2.421189	5.507710	0.009725	7	2.448120	5.604307	0.007809	7	2.456645	5.616607	0.007864
	6	2.948116	6.567176	0.011634	6	2.977733	6.661122	0.008368	6	2.982127	6.675379	0.008356
	1	3.427560	7.532147	0.013373	1	3.461297	7.626921	0.008880	1	3.461981	7.643216	0.008774
**17b**	1	-1.951967	0.633321	-0.003577	1	-2.012597	0.549853	0.025215	1	-2.010743	0.567343	0.014380
	32	-0.411842	0.712678	-0.000049	32	-0.478181	0.573044	0.003037	32	-0.476823	0.561250	-0.000648
	6	0.341319	-1.090160	0.000052	6	0.293617	-1.206051	0.001925	6	0.269111	-1.227668	-0.011398
	1	-0.009805	-1.637769	0.890339	1	-0.048763	-1.741989	0.902134	1	-0.087777	-1.765083	0.882285

	1	-0.006051	-1.636319	-0.892600	1	-0.071344	-1.750399	-0.884290	1	-0.098545	-1.758627	-0.904580
	1	1.438941	-1.045290	0.002398	1	1.390175	-1.154210	-0.012332	1	1.366355	-1.191965	-0.018011
	34	0.696034	2.580886	0.003583	34	0.628921	2.458296	-0.023262	34	0.662305	2.429293	-0.008117
	7	2.422661	5.504161	0.010287	7	2.462670	5.608706	-0.040225	7	2.553826	5.582783	-0.008892
	6	2.951088	6.565659	0.011955	6	3.040929	6.643109	-0.011966	6	3.087243	6.641210	0.006446
	6	3.605398	7.882987	0.014085	6	3.757690	7.925643	0.023418	6	3.747545	7.953912	0.026113
	1	4.573508	7.819745	-0.502392	1	3.242941	8.616303	0.705151	1	3.660059	8.394461	1.028604
	1	2.969763	8.616124	-0.502215	1	4.785162	7.763454	0.376516	1	4.809475	7.835346	-0.228661
	1	3.770736	8.215856	1.048407	1	3.783670	8.360874	-0.984859	1	3.268669	8.617403	-0.706776
**18a**	6	0.492073	-1.305186	-0.017162	6	0.482768	-1.342403	-0.000496	6	0.481785	-1.348040	-0.000481
	1	0.171328	-1.875557	0.870554	1	0.164158	-1.907626	0.890731	1	0.162587	-1.912420	0.891060
	1	0.177725	-1.868510	-0.911624	1	0.167252	-1.904849	-0.894574	1	0.165444	-1.910206	-0.894435
	1	1.585459	-1.201874	-0.012792	1	1.573960	-1.223085	0.001589	1	1.572913	-1.228456	0.001420
	32	-0.356705	0.457714	-0.012889	32	-0.402445	0.387847	0.000656	32	-0.404572	0.380496	0.000259
	6	-2.304064	0.267997	-0.020338	6	-2.340031	0.239711	-0.003203	6	-2.341748	0.239646	-0.003142
	1	-2.620809	-0.293971	-0.914806	1	-2.653133	-0.323911	-0.897340	1	-2.655791	-0.323642	-0.897122
	1	-2.626585	-0.301381	0.867372	1	-2.656459	-0.326490	0.888129	1	-2.658715	-0.325820	0.888430
	1	-2.783370	1.256150	-0.017747	1	-2.808361	1.232475	-0.002631	1	-2.806839	1.233872	-0.002687
	34	0.709430	2.352664	-0.000899	34	0.672135	2.299642	0.005753	34	0.672857	2.292800	0.004597
	7	2.407812	5.369785	0.017055	7	2.533259	5.487574	0.009296	7	2.540724	5.496798	0.009287
	6	2.989949	6.400083	0.021229	6	3.079718	6.535791	0.011437	6	3.083612	6.546756	0.011687
	1	3.519983	7.338124	0.025035	1	3.578663	7.493702	0.013407	1	3.579227	7.506594	0.013882
**18b**	6	0.479304	-1.328965	0.023258	6	0.440497	-1.414484	-0.020435	6	0.437602	-1.423172	-0.020418
	1	0.150825	-1.889035	0.914732	1	0.129835	-1.980330	0.873195	1	0.125340	-1.988958	0.872678
	1	0.173180	-1.903202	-0.867159	1	0.109960	-1.972401	-0.911929	1	0.106115	-1.979269	-0.912693
	1	1.572651	-1.225505	0.036142	1	1.532380	-1.302145	-0.032247	1	1.529541	-1.311633	-0.031714
	32	-0.369336	0.434684	-0.001398	32	-0.433541	0.321485	-0.003215	32	-0.435917	0.311911	-0.002044
	6	-2.316753	0.239132	-0.023990	6	-2.371972	0.184605	0.021615	6	-2.373985	0.184183	0.021346
	1	-2.623990	-0.334607	-0.914338	1	-2.701927	-0.374411	-0.869357	1	-2.704476	-0.372737	-0.870708
	1	-2.646072	-0.320282	0.867578	1	-2.678414	-0.382506	0.915862	1	-2.682521	-0.383069	0.914740
	1	-2.798777	1.225896	-0.037898	1	-2.834415	1.180083	0.032190	1	-2.832205	1.181559	0.032718
	34	0.694641	2.331115	-0.003162	34	0.652708	2.226658	-0.010805	34	0.655020	2.216535	-0.007813
	7	2.372795	5.325264	-0.000821	7	2.530829	5.411344	-0.011346	7	2.541021	5.416809	-0.010369
	6	2.949333	6.361504	0.006250	6	3.064802	6.469585	0.001329	6	3.071358	6.476837	0.001676
	6	3.664408	7.646819	0.015014	6	3.725717	7.782175	0.017283	6	3.727638	7.791593	0.016809
	1	3.599209	8.103701	1.012542	1	3.465414	8.314049	0.942566	1	3.466317	8.322353	0.942405
	1	4.721925	7.487654	-0.238702	1	4.814760	7.646641	-0.030899	1	4.817035	7.659517	-0.032547
	1	3.216754	8.328315	-0.722019	1	3.393462	8.372143	-0.847776	1	3.392213	8.380030	-0.848037
